# Contralateral delay activity during dynamic spatial updates in working memory

**DOI:** 10.1016/j.isci.2026.117916

**Published:** 2026-10-19

**Authors:** Charles Chernik, Ronald van den Berg, Mikael Lundqvist

**Affiliations:** 1 Division of Psychology, Department of Clinical Neuroscience, Karolinska Institutet, 171 77 Stockholm, Sweden; 2 Department of Psychology, Stockholm University, 106 91 Stockholm, Sweden; 3 The Picower Institute for Learning and Memory, Department of Brain and Cognitive Sciences, Massachusetts Institute of Technology, Cambridge, MA 02139, USA

**Keywords:** working memory, EEG, contralateral delay activity, attention, cognitive control

## Abstract

Working memory (WM) enables us to maintain and directly manipulate mental representations, yet we know little about the neural implementation of this privileged online format. We recorded electroencephalography data as human subjects engaged in a task requiring continuous updates to the locations of objects retained in WM as well as in a visually identical task with WM demands removed. Analysis of neural data suggested WM-related contralateral delay activity reversed polarity as objects held in WM moved to the opposite hemifield. The control task suggested this was partially but not fully explained by visual attention demands. Thus, the spatial location of activity related to both attention and WM was updated to meet behavioral demands as the spatial location of remembered objects changed.

## Highlights

• Contralateral delay activity tracks mental movement of working memory representations
• CDA polarity is reversed when memorized objects move to the opposite hemifield
• CDA polarity reversal is not fully explained by visuospatial attention

## Graphical abstract

**Figure:**
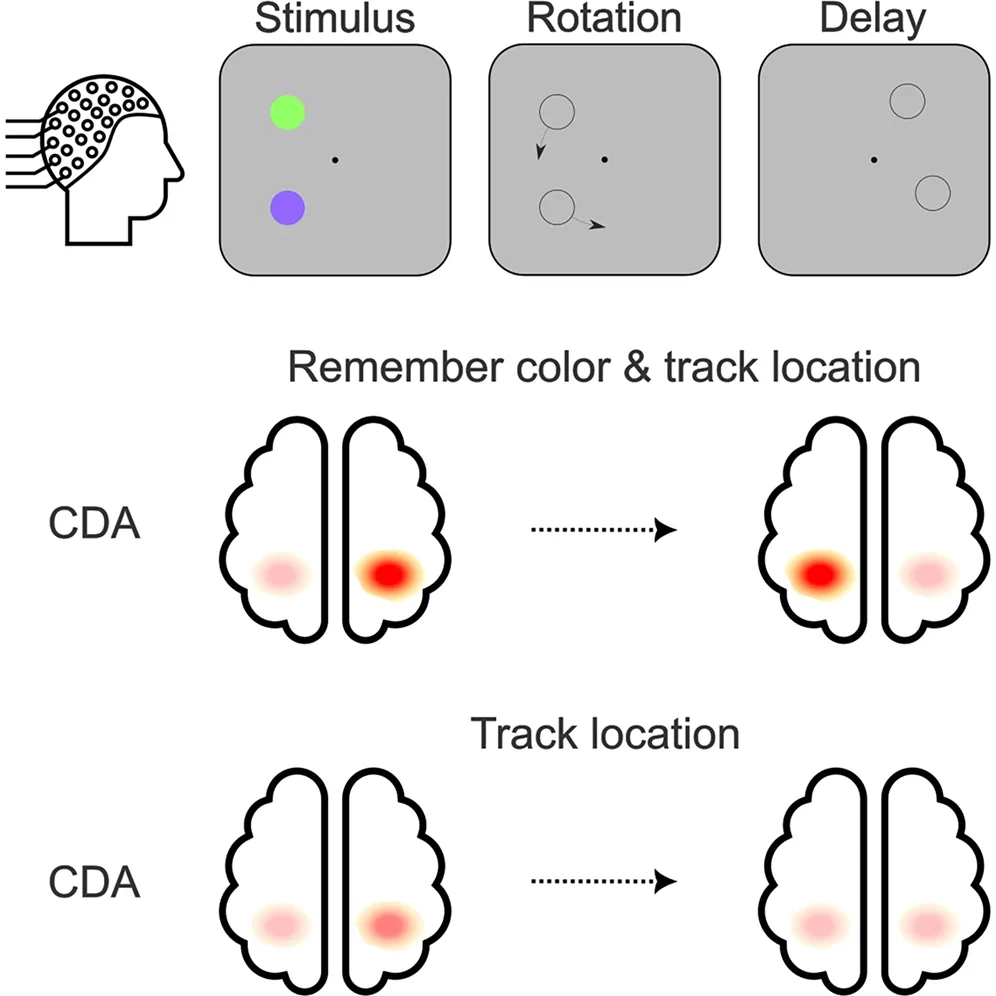

## Teaser

Biological sciences; Cognitive neuroscience; Natural sciences; Neuroscience

## Introduction

A fundamental aspect of cognition is our ability to hold and mentally manipulate information in an online state in working memory (WM).^1^^,^^2^ Both the format of retention^3^^,^^4^^,^^5^ and the actual manipulation of WM information^6^^,^^7^^,^^8^^,^^9^^,^^10^ are highly studied topics. Despite this, we know relatively little about the transformations of memory representations that underlie such manipulations.^11^ This knowledge gap may in part be due to WM representations typically being studied under relatively static settings, while in real-life WM representations are continuously updated to support behavior.^12^^,^^13^

Spiking and local-field potential data from monkeys suggest that the representation of visual objects transfers from one prefrontal hemisphere to the other following saccades that move the location of an earlier presented stimulus to the opposite hemifield.^12^ Likewise, gaze bias in humans, a behavioral correlate of the location of retained WM objects, shifts as subjects turn their heads.^13^ These findings suggest that WM object representations transfer across cortex when humans mentally update their remembered locations, but electrophysiological evidence for this is lacking. Here, we aim to bridge that gap by using a novel experimental paradigm combined with electroencephalogram (EEG) measures.

We tracked the hemispheric locations of WM representations using contralateral delay activity (CDA), an electrophysiological correlate of WM representations measured via EEG.^14^^,^^15^ This measure reflects the difference in brain activity between the electrodes contralateral and ipsilateral to the side where the remembered items were presented. It correlates with the number of relevant but not irrelevant visual WM objects, and is often used as a correlate of lateral WM load.^14^ Prior work has shown that CDA is sensitive to the selection of a subset of particular items after initial encoding,^16^ as well as subsequent encoding of additional items.^17^ Due to its lateralized nature, CDA offers a way to infer whether memory representations are transferred between hemispheres when remembered stimuli are mentally relocated from one visual hemifield to the other. Here, we have tested this by recording EEG while participants performed a WM task that required them to mentally update the locations of two remembered items across visual hemifields. Our main hypothesis was that when the remembered locations of stimuli move across visual hemifields, the underlying neural representations likewise relocate across hemispheres, reversing the sign of the CDA signal.

## Results

### Experiment 1: WM

We recorded EEG from healthy humans during a delayed-estimation task^18^ that involved mental updating of locations of memorized stimuli after they disappeared from the screen (Figure 1A). Each trial began with a central fixation, followed by a cue indicating on which side (left or right of the fixation point) the to-be-remembered objects would appear. Four differently colored discs (two on each side) were then shown, with the two non-cued items serving to balance visual inputs in the two hemispheres and ensuring activity differences were due to memory-related processes. After 500 ms, the four discs were replaced by colorless outlines that started moving 1 s later along an (invisible) circle (180°/s, clockwise or counterclockwise, 0°–360°, with 13 possible distances; Figure 1B). Finally, 3 s after movement onset, one of the outlines was highlighted and the subject was prompted to provide their estimate of its remembered color through a mouse click on a color wheel. Importantly, since the direction and degree of movement were unpredictable, accurate performance was possible only if the subjects mentally updated item positions in WM in real time.

**Figure 1:**
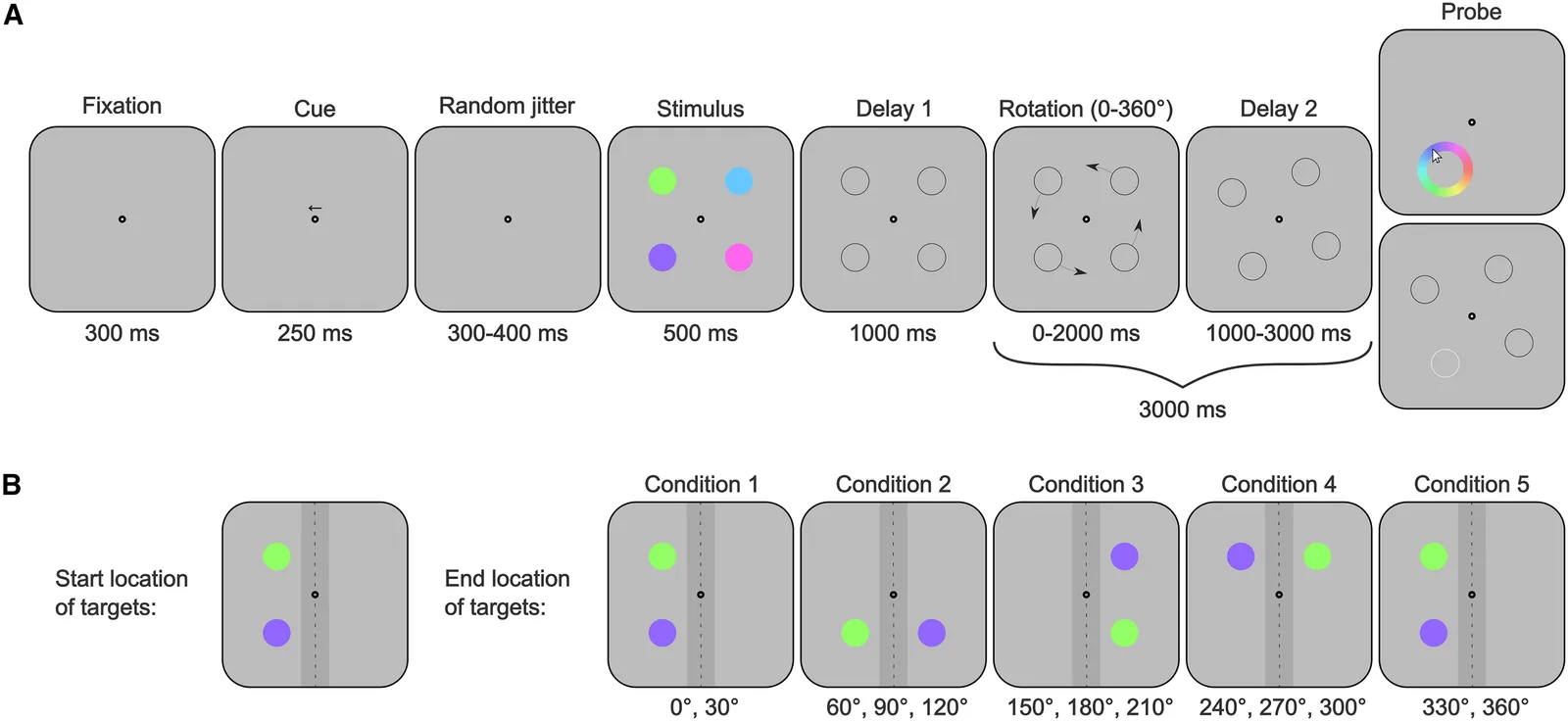
Task design of the experiments (A) Trial structure. Each trial started with a fixation period of 300 ms, followed by a cue indicating the to-be-attended side (left or right). After a random jitter of 300–400 ms, a memory array of four colored discs appeared, with two on each side of the fixation point. After 500 ms, the colored discs were replaced with black outlines to indicate their locations. After 1,000 ms the outlines simultaneously started moving along an invisible circle, changing their positions relative to the midline by 0°, 30°, …, or 360° (13 possible alternatives, with randomized sign, 180°/s). Once the movement was complete, the outlines remained in their new positions for at least 1 s. Then, in Experiment 1 (top), all the outlines disappeared, and a color wheel was presented at the post-movement location of one of the memorized discs, prompting recall. The response was submitted via a mouse click anywhere on the color wheel. In Experiment 2 (bottom), the outlines stayed in place, with one of them changing its color to white. The subject indicated through a keyboard button press whether the white outline corresponded to one of the tracked discs. (B) Schematic illustration of the five experimental conditions, defined by the laterality of the post-movement stimulus locations: Condition 1—both discs remained in the original hemifield; Condition 2—one of the discs transferred to the other hemifield; Condition 3—both discs transferred to the other hemifield; Condition 4—both discs transferred to the other hemifield and then one of them returned to the original hemifield; and Condition 5—both discs transferred to the other hemifield and then returned to the original one. While there were 13 possible rotation angles, each of them fell in one of these five conditions. The dark gray region represents the area along the midline (width = 1.6°), where the discs could not end up after the movement, ensuring lateralization.

The response error with respect to the target item gradually increased with larger movement angles (Figure 2A, green), while the error with respect to the non-target item (computed the same way but for the cued item that was not probed) decreased (Figure 2A, purple). This suggests that subjects tended to confuse the two retained colors (swapping errors^19^) more often with larger movements. This was corroborated by fitting the data with an equal-precision model with non-target responses,^20^ which explained the performance degradation through increasing swapping rates at larger movements, while memory precision of the two items remained stable (Figure 2B). Importantly, even at the largest movement angle subjects performed accurately: estimated swap rates were relatively low (around 10%) and memory precision was high (parameter log(*κ*) was on average around 2, which corresponds to an average memory error of 16.8° on non-swap trials). Hence, participants must have been updating item position in WM in real time.

**Figure 2:**
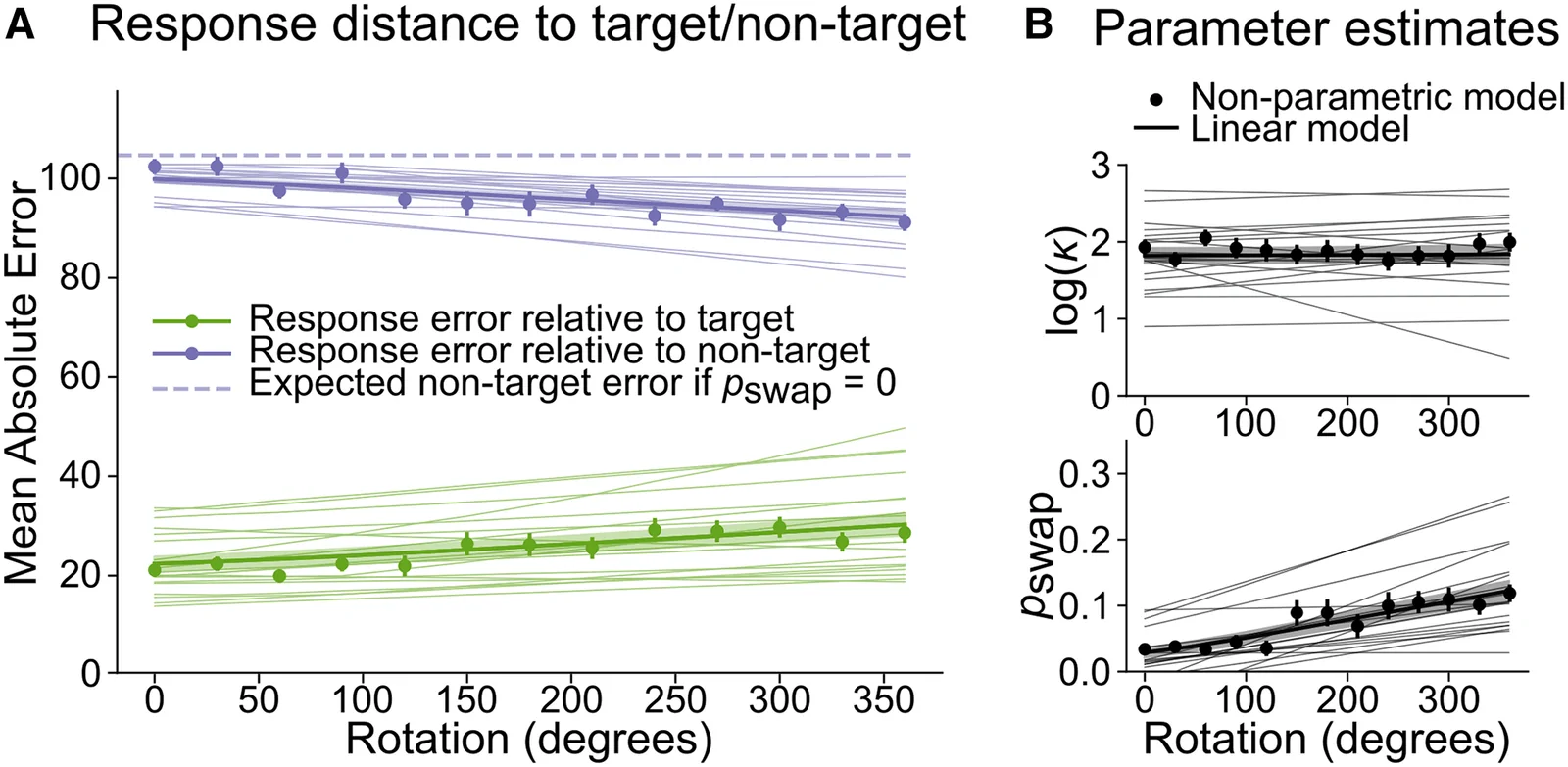
Effect of stimulus movement on response accuracy (A) Subject-averaged response errors as a function of movement angle. The error relative to the target (green markers) gradually increased with movement angle (Pearson’s *r* = 0.88, *p* < 0.001), while the error relative to the non-target item (purple markers) decreased (*r* = −0.89, *p* < 0.001). These trends were well accounted for by a model in which memory precision and swap rate are linearly related to motion angle (thin lines: estimates for individual subjects; thick lines: averages). Shaded areas and error bars represent one SEM. (B) Estimated effect of movement angle on memory precision (top) and swap rates (bottom) in the linear model (lines) and in a non-parametric model (markers). While swap rate increased (*r* = 0.92, *p* < 0.001), there was no evidence that memory precision was affected by motion angle (*r* = −0.02, *p* = 0.93).

Next, we computed the CDA from the EEG data to investigate the neural underpinning of this mental updating. Under the hypothesis that mental updating of item locations caused WM representations to transfer between hemispheres (Model 1), both the magnitude and sign of the CDA were predicted to change in specific ways as the empty discs moved across visual hemifields (Figure 3A): right before motion onset, the CDA should be negative, as in previous studies using stationary items; in trials where both discs stayed in the original hemifield (Condition 1), the CDA should remain negative; if one disc moved over to the other hemifield (Condition 2), the CDA should disappear, because the two WM objects would be in opposite hemispheres, eliminating the lateralized imbalance that drives the CDA; if both discs crossed the midline (Condition 3), the CDA should change sign to positive; if one disc then moved back into the original hemifield, the CDA should again disappear (Condition 4); finally, in the trials where both discs moved full circle (Condition 5), the post-motion CDA should be negative again, as if no rotation had taken place.

**Figure 3:**
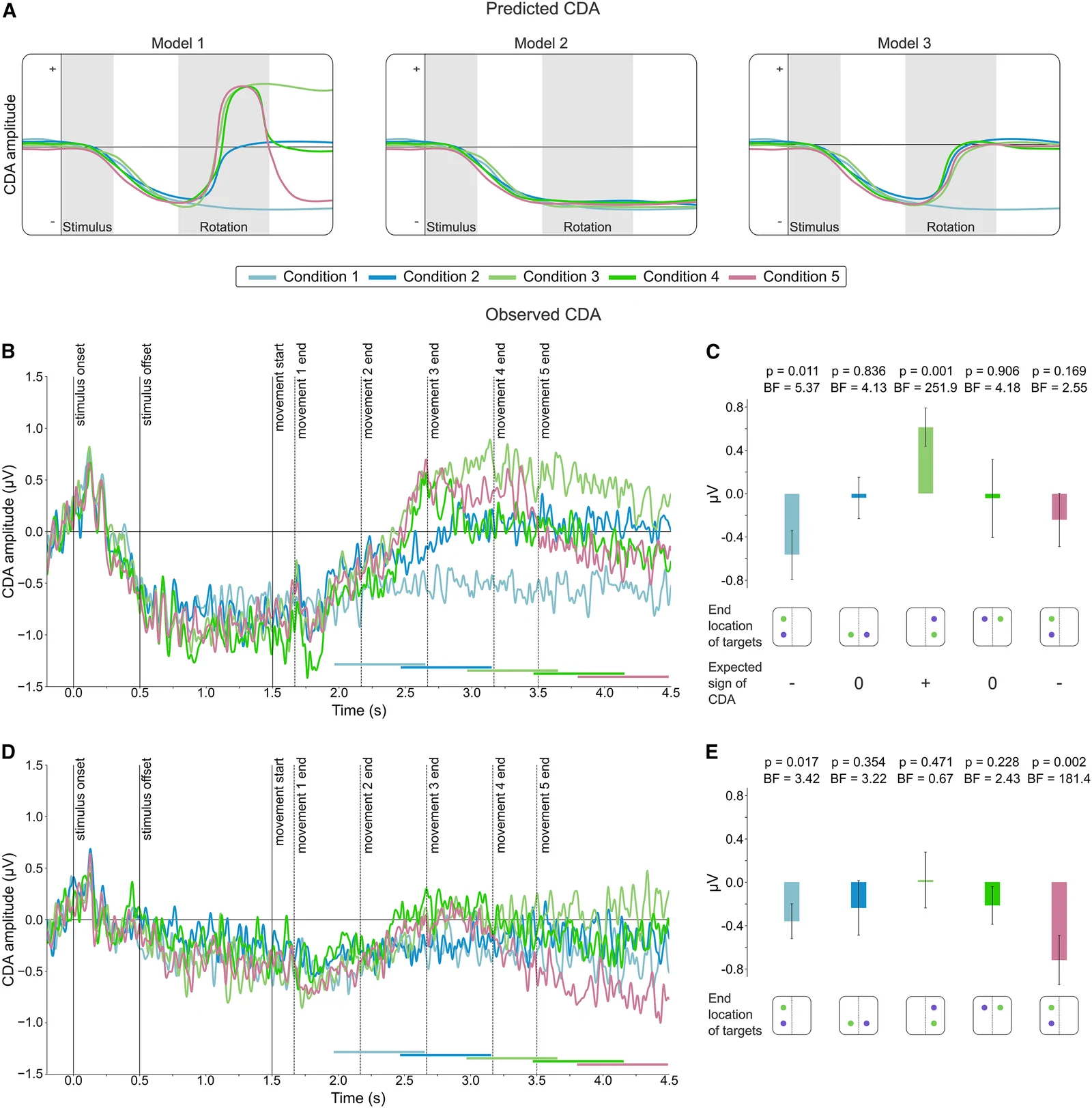
CDA following movement of WM objects/attentional tracking (A) Cartoon of model predictions of how the CDA changes following the mental relocation of the discs. In the main model, Model 1, the CDA changes depending on the hemifield the discs end up in post rotation (Condition 1–5). For Conditions 1 and 5, the discs end up in the initial hemifield and the CDA should be negative. For Conditions 2 and 4, there is one disc in each hemifield and there should be no CDA. For Condition 3, both discs end up in the opposite hemifield and the CDA should be positive. As alternatives, Model 2 predicts that the neural representations of the disks remain in their original locations (meaning the CDA remains unchanged) and Model 3 predicts the CDA disappears completely after the update of WM contents occurs (meaning the CDA only remains when both discs stay in the initial hemifield). (B) Measured CDA per condition in Experiment 1. Dotted vertical lines denote the latest possible time when movement ended per condition. Colored horizontal bars denote 300–1,000 ms post movement per condition, used to analyze the CDA statistically. (C) Top: average CDA post movement per condition in Experiment 1, taken from the time periods indicated in (B). Error bars denote SEM. The *p* values were obtained from *t* tests on the null hypothesis of CDA being 0. In the conditions where Model 1 predicted a specific effect direction, one-sided tests were used. The BFs were obtained from Bayesian *t* tests and quantify the evidence in favor of model 1 (see STAR Methods). Middle: cartoon demonstrating the end location of the targets per condition (assuming both discs started on the left). Bottom: sign of the CDA predicted by Model 1. (D) Measured CDA per condition in Experiment 2. (E) Average CDA post movement per condition in Experiment 2.

To our knowledge, there is currently no prior CDA-focused literature that makes explicit alternative predictions about how mental relocation of memoranda across visual hemifields should affect CDA polarity or amplitude. Therefore, we formalized two benchmark hypotheses against which we tested our main hypothesis. One alternative hypothesis is that the neural representations of WM objects remain anchored to their original encoding locations, regardless of subsequent mental updates of spatial positions (Model 2). This model predicts that the sign of the CDA remains negative throughout any post-encoding updates of locations, with a magnitude that is either stable or may gradually decrease to reflect decaying memory quality. A final hypothesis is that mental movement turns the neural representations into a new format that is not tracked by CDA (Model 3). This model predicts that cross-hemispheric movements cause CDA (possibly apart from Condition 1) to disappear and not come back.

Qualitatively, our data closely aligned with the predictions of Model 1, with the CDA starting off negative, reversing sign as WM objects transitioned into the opposite hemifield and then again when they moved back into the original hemifield (Figure 3B; see also Figure S1A for the contralateral and ipsilateral waveforms without subtraction).

To test the predictions of Model 1 quantitatively, we performed *t* tests on the time-averaged CDA in each of the five conditions, in a 700 ms window shortly after movement ended in each condition (Figure 3B, horizontal bars). For all 5 conditions, the Bayes factors (BFs) supported the prediction of Model 1 (Figure 3C, *n* = 19 in all tests; see also Figure S2 for the CDA amplitude distributions), though for Condition 5, the evidence was anecdotal (BF = 2.55). Similarly, the *p* values were in line with the model’s predictions for all conditions except Condition 5. These results were robust against changes in how the CDA time windows were defined (Figure S3 and supplemental information). In the three conditions where Models 1 and 2 differed in their predictions, the data favored Model 1, with BFs of 3.57 (Condition 2), 471.7 (Condition 3), and 3.84 (Condition 4). In the comparison between Models 1 and 3, Model 1 was clearly favored in Condition 3 (BF = 32.1), but the evidence was weakly in favor of Model 3 in Condition 5 (BF = 0.60).

To compare the models more directly, we fitted Bayesian mixed-effects models corresponding to the three theoretical accounts. Model comparison favored Model 1, which received 70.9% of the model weight, compared with 18.5% for Model 2 and 10.6% for Model 3. Model weights reflect the relative predictive contribution of each model to explaining the data. In Model 1, the estimated coefficients were −0.56 μV for Condition 1 (95% highest density interval, HDI [−1.03, −0.06]), 0.60 μV for Condition 3 (95% HDI [0.10, 1.06]), and −0.33 μV for Condition 5 (95% HDI [−0.72, −0.001]), whereas Conditions 2 and 4 were constrained to zero. Thus, the posterior estimates for the population-level parameters closely matched the predicted hemispheric-transfer pattern of Model 1.

Finally, the results of a coarse exploratory source-level analysis (Figure S4 and supplemental information) were compatible with the hypothesized hemispheric relocation of activity, showing evidence for a reversed modulation in Condition 3 compared to Conditions 1 and 5 (odds ratio for reversal: 14.0).

### Experiment 2: Attentional tracking

While the CDA is widely regarded as a neural marker of WM storage,^15^^,^^21^^,^^22^ some studies suggest that it is also sensitive to attentional processes.^23^^,^^24^^,^^25^ To test to what extent the patterns observed in Experiment 1 reflect attentional processes rather than WM retention, we conducted another experiment with the same sequence of stimuli as in Experiment 1, but with a task that did not require storing them in WM. In Experiment 2, participants were instructed to ignore the colors and simply keep track of the positions of the two cued discs. At probe, one of the four disks was highlighted, and, instead of recalling its color, participants indicated whether it was one of the two tracked discs or not. If the hemispheric transfer suggested by the CDA patterns in Experiment 1 was primarily a marker of attentional tracking, we expected the pattern in Experiment 2 to be similar, both qualitatively and quantitatively. However, if the transfer of WM contents was an important driver of the patterns observed in Experiment 1, then the CDA and sign reversals should be less pronounced in Experiment 2.

As this was a simple task, the behavioral performance was unsurprisingly good (accuracy = 94.2% ± 5.1%, M ± SD). Since the task in Experiment 2 did not involve WM encoding, we first checked if the stimuli evoked a CDA signal at all, by performing a Bayesian *t* test on the CDA in the 700 ms window before the movement (this part of the trial was identical across the five conditions). Evidence for a negative CDA was observed (BF = 22.7), albeit of much weaker strength than in Experiment 1 (BF = 141.03). Hence, the CDA in our paradigm appears to reflect a mixture of attentional and WM processes, with attention alone producing a weaker and less consistent signal.

Next, we computed and analyzed the post-movement CDA the same way as before. Qualitatively, the observed pattern was comparable to the one in Experiment 1 (Figure 3D; see also Figure S5 for the contralateral and ipsilateral waveforms without subtraction), yet there were marked quantitative differences. Notably, unlike in Experiment 1, no polarity reversal from negative to positive was observed in Condition 3 (Figure 3E; see also Figure S6 for the CDA amplitude distributions).

Bayesian mixed-effects model comparison yielded 52.2% model weight for Model 1, 47.8% for Model 2, and a negligible weight for Model 3. The estimated coefficients in Model 1 were −0.35 μV for Condition 1 (95% HDI [−0.67, 0.00]), 0.22 μV for Condition 3 (95% HDI [0.00, 0.54]), and −0.66 μV for Condition 5 (95% HDI [−1.11, −0.21]). In Model 2, the estimated intercept was −0.30 μV (95% HDI [−0.52, −0.09]). In contrast to the WM experiment—where Model 1 was clearly favored over the other models—model comparison in the attention experiment was inconclusive, with Models 1 and 2 receiving nearly equal support.

In sum, the CDA results of Experiment 2 provided weaker and less consistent support for the hemispheric-transfer account when WM was not engaged. Although some initially lateralized activity resembling CDA was present, the critical polarity reversal predicted by Model 1 was not clearly observed and model comparison did not clearly favor the transfer model over the alternative account in which CDA remains anchored to the originally attended location.

Finally, the exploratory source-level analysis (Figure S4 and supplemental information) showed no clear pattern of relocation of activity between conditions and, compared to Experiment 1, provided only weak evidence for a reversal in Condition 3 compared to Conditions 1 and 5 (odds ratio: 3.1:1).

### Polarity reversal in combined Conditions 3–5

To further investigate the observed CDA polarity reversals, we took advantage of the fact that the midline crossing occurs in three out of five conditions (Conditions 3, 4, and 5) and combined the data across these conditions to obtain higher statistical power (as the number of trials effectively tripled compared to single-condition analyses). We then calculated the CDA for the resulting “combined” condition and ran a one-sided cluster-based permutation test to detect whether it was significantly above zero following the movement in either experiment. We focused the analysis on the last 2.5 s of the trial: from approximately 250 ms before the time the second outline crossed the midline until the end of the trial.

For Experiment 1, two significant clusters were identified (Figure 4A). In contrast, no positive clusters were found for Experiment 2 (Figure 4B). As the contributions of each condition to the combined CDA were essentially equal (∼1/3 of the trials), this suggests that evidence of CDA reversal in Experiment 1 was present not only in Condition 3, as indicated by the condition-wise analyses reported previously, but also in Conditions 4 and 5.

**Figure 4:**
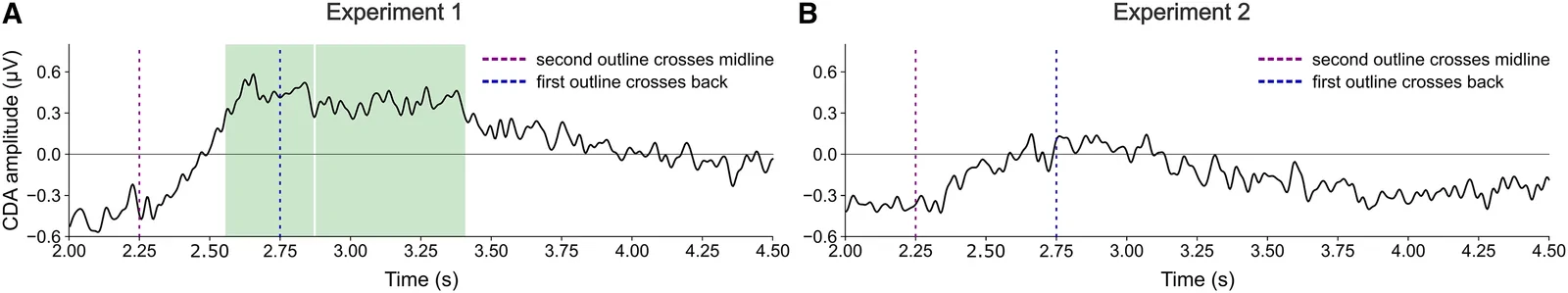
CDA for combined Conditions 3–5 (A and B) CDA computed by pooling the data from Conditions 3-5 for (A) Experiment 1 and (B) Experiment 2. Green shading indicates the time points at which the CDA was significantly above zero according to a cluster-based permutation test (one-sided, *p* < 0.05). Purple dotted line indicates the time when the second outline crosses the midline (in all three conditions), blue dotted line indicated the time when the first outline crosses the midline back into the original hemifield (in Conditions 4 and 5).

### Comparison of CDA between experiments

To directly compare the two experiments, we conducted an additional Bayesian hierarchical analysis of CDA amplitudes across experiments and rotation conditions. In Condition 3, where the memorized items moved to the opposite hemifield, the CDA was larger in Experiment 1 than in Experiment 2 (mean difference = 0.59 μV, 95% HDI [−0.05, 1.26]). The posterior odds ratio was 27.1:1 in favor of a positive difference, indicating that a larger CDA in Experiment 1 was about 27 times more likely than the opposite pattern (i.e., a larger CDA in Experiment 2). We further quantified the magnitude of the CDA reversal relative to Conditions 1 and 5. The reversal profile was positive in both experiments, with strong evidence in Experiment 1 (mean = 1.03 μV, 95% HDI [0.43, 1.63], odds ratio = 1332:1) and in Experiment 2 (mean = 0.57 μV, 95% HDI [0.05, 1.08], odds ratio = 58.0:1). The between-experiment comparison suggested that this modulation was larger in Experiment 1 than in Experiment 2 (mean difference = 0.46 μV, 95% HDI [−0.38, 1.21]), with posterior odds of 7.3:1 in favor of a stronger modulation in Experiment 1.

These results indicate that although attentional tracking alone can produce lateralized activity resembling CDA, it does not reproduce the full pattern observed in Experiment 1. In particular, the polarity reversal in Condition 3 was absent in Experiment 2, and overall evidence for modulation of the CDA pattern was weaker, consistent with the interpretation that maintaining items in WM enhanced the magnitude of the modulation.

### Analysis of lateralized alpha power

To assess whether the observed CDA effects might reflect oscillatory dynamics associated with spatial attention, we conducted additional analyses of lateralized alpha power. First, we repeated the same within-experiment analyses as we performed for the CDA. The observed patterns were both qualitatively (Figures 5A and 5C) and quantitatively (Figures 5B and 5D) similar between the two experiments, with alpha lateralization following the shifting focus of spatial attention.

**Figure 5:**
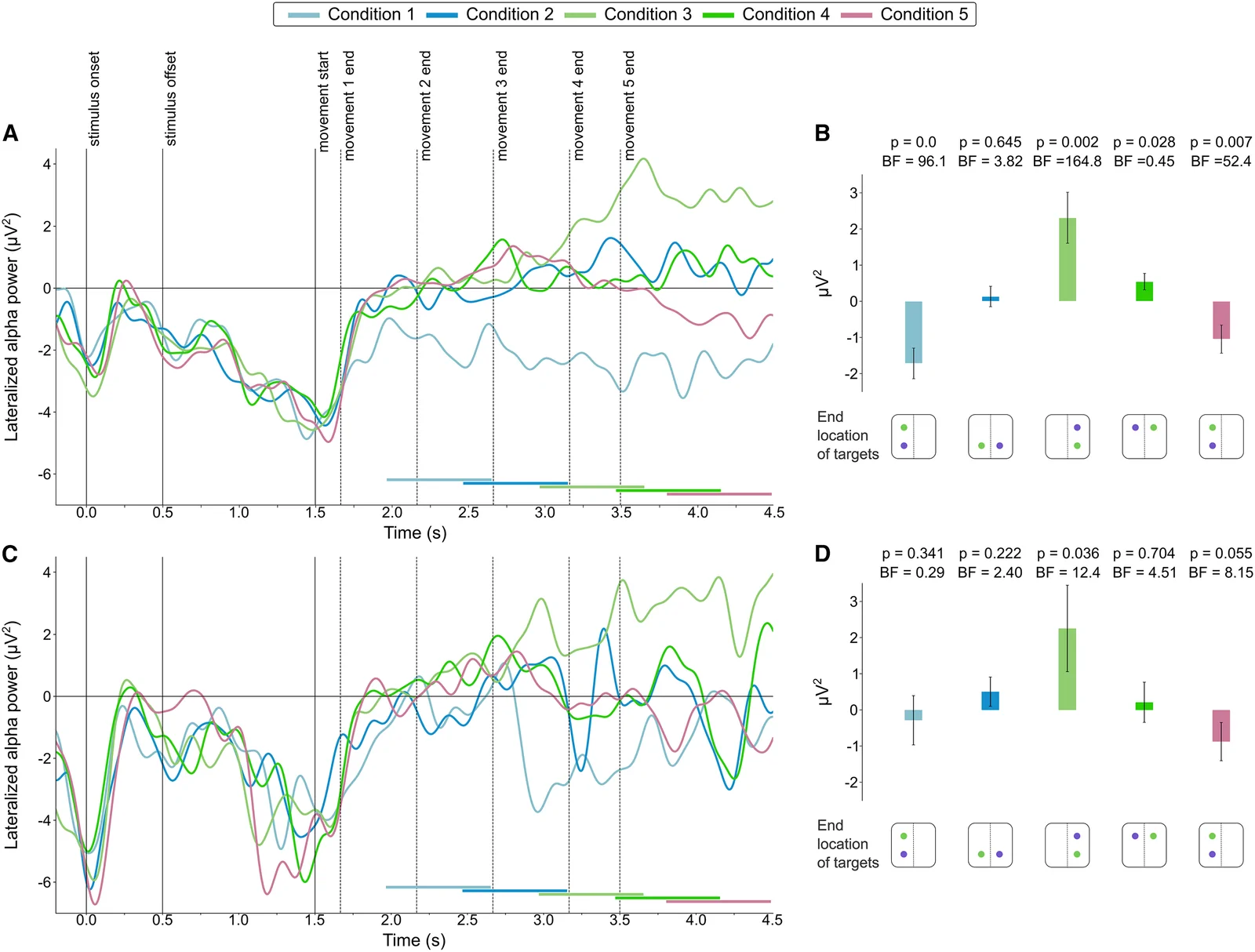
Lateralized alpha power following movement of WM objects/attentional tracking (A) Lateralized alpha power per condition in Experiment 1. Dotted vertical lines denote the latest possible time when movement ended per condition. Colored horizontal bars denote 300–1,000 ms post movement per condition, used to analyze the lateralized alpha power statistically. (B) Top: average lateralized alpha power post movement per condition in Experiment 1, taken from the time periods indicated in (A). Error bars denote SEM. The *p* values and BFs were obtained from *t* tests with the hypotheses defined the same way as in the corresponding CDA analyses (see Figure 3 and STAR Methods). Bottom: cartoon demonstrating the end location of the targets per condition (assuming both discs started on the left). (C) Lateralized alpha power CDA per condition in Experiment 2. (D) Average lateralized alpha power post movement per condition in Experiment 2.

For Experiment 1, the pattern of lateralized alpha activity thus also closely resembled that of the CDA, although, consistently with prior research,^26^^,^^27^ alpha power was slower to lateralize. For Experiment 2, however, there was a marked difference between the CDA and alpha lateralization patterns in Condition 3: significant polarity reversal after the movement was observed in lateralized alpha that was not present in the CDA. Interestingly, in Condition 1, alpha power became less lateralized following the movement end, but then relateralized later in the trial (Figure 5C). A potential explanation for this pattern, as well as its absence in Experiment 1, is that in the attentional tracking task, when the movement was minimal or absent, the subjects did not need to maintain continuous focus for the entire ∼3 s delay and instead refocused later in anticipation of the probe.

For a direct between-experiment comparison, we quantified modulation of alpha power across rotation conditions in both experiments using the same hierarchical modeling framework as in the previous section. Alpha power showed clear modulation across conditions in both tasks. In Experiment 1, the alpha profile modulation relative to Conditions 1 and 5 was strongly positive (mean = 3.73 μV^2^, 95% HDI [1.19, 6.34], odds ratio = 355:1). A similar modulation was observed in Experiment 2 (mean = 2.81 μV^2^, 95% HDI [0.76, 4.97], odds ratio = 275:1). Importantly, the magnitude of this modulation did not meaningfully differ between experiments (mean difference = 0.92 μV^2^, 95% HDI [−2.32, 4.37], odds ratio = 2.45:1). Likewise, there was essentially no evidence for a Condition 3 difference between experiments (mean difference = 0.10 μV^2^, 95% HDI [−3.20, 3.19], odds ratio = 1.09:1).

Thus, alpha-band power tracked the spatial manipulation similarly in both experiments—consistent with the engagement of spatial attention and tracking processes—but it did not reproduce the stronger CDA polarity reversals observed in Experiment 1. This dissociation indicates that the critical CDA effects cannot be attributed solely to oscillatory alpha dynamics associated with attentional tracking and likely reflect additional processes related to the maintenance and updating of WM representations.

### Gaze bias analyses

Finally, to verify that the observed differences in CDA patterns between the two experiments were not driven by differences in systematic eye movements, we analyzed horizontal gaze bias during the post-movement delay. While there was significant horizontal bias in the direction of the post-movement location of the stimuli, it did not differ significantly between experiments (Figure S7 and supplemental information). We also found no evidence that gaze bias predicts CDA amplitude in any condition in Experiment 1 and four out of five conditions in Experiment 2 (see Figure S8).

## Discussion

To gain insight into the neurophysiological processes underlying real-time manipulations of WM representations in a dynamic world, the current study examined the CDA—an electrophysiological marker of WM load—in a novel experimental paradigm where subjects were required to encode objects (colored discs) into WM and then update the spatial location associated with them. In a separate experiment, subjects completed a visually identical task without having to remember the colors. Together, the results suggested that dynamic updates to the relative location of objects held in mind are reflected in qualitative changes in the CDA. These changes were related to both the updated WM information and attentional processes.

In Experiment 1, we observed that the CDA followed the mental movement in real time, reversing its sign when the objects moved into the opposite hemifield, and then again when they returned to the original hemifield. These results were consistent with our main hypothesis that mentally updating the location of a memorized item causes a hemispheric transfer of the neural representation of that item, although it should be noted that the evidence for Condition 5 was relatively weak. Interestingly, our findings stand in contrast to those of an earlier study that found no CDA sign reversal when objects were encoded in one hemifield and later tested in the opposite one.^28^ We suspect the effect observed here requires a task with strong emphasis on updating contents in real time to prevent the updating during the test period itself. Consistently, including subjects with poor accuracy on the behavioral task reduced the effect (Figure S9). However, the source of the effect could also be the continuous visuospatial attention required in our task, if CDA is driven by attention rather than WM. The aim of Experiment 2 was to examine to what extent attentional tracking can explain the effect.

The results from Experiment 2 suggested that neither interpretation alone could fully account for the dynamically updated CDA. If the CDA effects observed in Experiment 1 were solely due to attentional tracking of moving placeholders, then similar CDA patterns should have emerged even in the absence of a WM requirement. Conversely, if the CDA strictly reflected maintained and manipulated WM content, no sustained lateralized activity would be expected when subjects attended to the placeholders without being instructed to encode or store color information.^17^^,^^26^ Instead, the observed CDA response in Experiment 2 showed partial activation, suggesting that attentional tracking alone may elicit some CDA-like activity, but the full effect seen in Experiment 1 likely depends on the presence of active WM representations. Crucially, unlike in Experiment 1, the observed CDA did not reverse polarity in any condition at any time point, indicating that polarity reversal as the items cross between hemifields could be a distinctive marker of active updating of spatial WM information.

Another interesting observation was the strong negative post-movement CDA in Condition 5 of the attention task. This strong negative CDA, compared with the relatively weak and inconclusive effect in the same condition in the WM task, represents a notable divergence between the two experiments. One tentative explanation is that this difference relates to the absence of a polarity reversal in the attention task. If attentional tracking remained anchored to the original hemifield even when both discs crossed the midline, then when they returned in Condition 5 the CDA would re-emerge strongly negative, unlike in the WM task where intermediate remapping attenuated the effect. Further work will be needed to clarify the mechanisms behind the divergence in Condition 5.

Importantly, the differences between the experiments observed in the CDA were not present in either lateralized alpha activity, an established marker of spatial attention,^22^^,^^26^^,^^29^ or systematic eye movements. Hence, attentional and oculomotor processes cannot fully account for the differences in the CDA, suggesting that these differences were instead driven by WM processes.

Previous research has shown CDA to reflect filtering (tracking the number of relevant items, and not distractors^14^^,^^21^) and to dynamically track various transformations of the WM load—such as grouping,^30^^,^^31^ splitting,^32^ and sequential encoding,^17^—but its response to spatial manipulations, up until very recently,^33^ has been explored only in the context of multiple-object tracking (MOT).^24^ Notably, Drew et al.^24^ observed CDA reversing polarity in their MOT task, which they refer to as “handoff of attention.” However, the lack of such a reversal in our second experiment contradicts this purely “attentional” interpretation. Additionally, previous findings suggest that object-tracking-related CDA fades approximately 350 ms after the objects stop moving,^23^ whereas we observed sustained CDA throughout the post-movement delay periods with objects held fixed, suggesting WM- rather than attention-related activity. What is more, a recent study by Wang et al.^34^ suggests that the classic MOT tasks may include an implicit WM demand by requiring participants to maintain the spatial representations of the tracked stimuli in WM, meaning that past observations from MOT might have reflected more than just attentional processes too.

In both WM and MOT literature, CDA has widely been considered to reflect item-based processes, be it WM maintenance or attentional tracking. Recently, a refined view has emerged, suggesting that it reflects a common process of assigning spatiotemporal “pointers.”^35^ In a combined WM and MOT task, Styrkowiec et al.^33^ observed CDA reflecting tracking load rather than WM load when both were simultaneously tested. The authors interpreted this as evidence that CDA reflects an indexing mechanism rather than content-related activity. However, this view cannot account for the observed differences between our two experiments. Even if the two disks in Experiment 2 were assigned a single pointer (which could explain a weaker pre-movement CDA compared to Experiment 1), it would not explain the lack of polarity reversal during or after the movement. Moreover, the pointer-based accounts are difficult to reconcile with the growing body of evidence showing CDA to reflect more than discrete item-based processing,^36^ as it has been observed to be sensitive to perceptual complexity,^37^^,^^38^ semantics,^39^^,^^40^ and familiarity.^41^ Our results seem in line with these less discrete accounts of the nature of CDA.

In conclusion, our results are consistent with hemispheric transfer of WM-related activity when remembered objects moved between hemifields. This would put them in line with single neuron activity recorded from non-human primates suggesting that neural information retaining WM object identity is transferred across hemispheres as item locations are updated.^12^

### Limitations of the study

The main limitations of the study stem from the inherently low signal-to-noise ratio of scalp EEG recordings and the deep-rooted difficulties in disentangling attention and WM processes. The nature of the CDA is still debated: it is unclear to what extent the observed activity reflects attentional mechanisms, pointers (or a similar indexing mechanism), or the WM content itself. Whether it was the actual WM content that was transferred could be further tested by using methods directly decoding object identity from EEG.^42^ We deployed two distinct tasks to disentangle WM from attention, but while the attention task did not formally require WM, it is still possible that the subjects partially remembered the colors of the discs or engaged spatial WM during the tracking. Such an implicit WM demand would reduce the expected difference in CDA between the two tasks and potentially explain the dampened CDA effects in our attention task rather than them being attributable to attention. Decoding WM contents has the potential to rule out this possibility as well.

## Resource availability

### Lead contact

Requests for further information and resources should be directed to and will be fulfilled by the lead contact, Charles Chernik (charles.chernik@ki.se).

### Materials availability

This study did not generate new materials.

### Data and code availability

• Preprocessed and epoched EEG data and associated behavioral data have been deposited at an Open Science Framework (OSF) repository and are publicly available as of the date of publication. The link to the repository is listed in the key resources table. Raw data are available from the lead contact upon reasonable request.
• All original analysis code has been deposited at an OSF repository and is publicly available as of the date of publication. The link is listed in the key resources table.
• Any additional information required to reanalyze the data reported in this paper is available from the lead contact upon request.

## Acknowledgments

This work was funded by 10.13039/100017325ERC starting grant 949131 and Swedish research council project grant VR 2022-02328.

## Author contributions

Conceptualization, C.C., R.v.d.B., and M.L.; data curation, C.C.; formal analysis, C.C. and R.v.d.B.; funding acquisition, M.L.; investigation, C.C.; methodology, C.C., R.v.d.B., and M.L.; software, C.C. and R.v.d.B.; supervision, M.L.; visualization, C.C.; writing – original draft, C.C., R.v.d.B., and M.L.; writing – review and editing: C.C., R.v.d.B., and M.L.

## Declaration of interests

The authors declare no competing interests.

## STAR★Methods

### Key resources table

**Table:** 

REAGENT or RESOURCE	SOURCE	IDENTIFIER
**Deposited data**		

Preprocessed and epoched EEG and behavioral response data	This paper	OSF: https://doi.org/10.17605/OSF.IO/SHJV8

**Software and algorithms**		

Analysis scripts	This paper	OSF: https://doi.org/10.17605/OSF.IO/SHJV8
Psychtoolbox	The Psychophysics Toolbox, https://psychtoolbox.org/	RRID: SCR_002881
MNE-Python	Gramfort et al.^43^, https://mne.tools/stable/install/index.html	RRID: SCR_005972
JASP	JASP Team (2024), https://jasp-stats.org/	RRID: SCR_015823

### Experimental model and study participant details

#### Participants

A total of 26 (Experiment 1, fall 2023) and 31 (Experiment 2, summer-fall 2024) subjects were recruited via Karolinska Institutet’s online recruitment system. All subjects provided informed written consent before the start of the experiment and were reimbursed 100 SEK per hour. The study was approved by the Swedish Ethical Review Authority (Dnr. 2022-05444-01) and was performed according to the Declaration of Helsinki. All subjects reported normal or corrected-to-normal visual acuity and no neurological disorders. Prior to participation in Experiment 1, color vision was tested using the short (6-plate) version of the Ishihara’s Test for Color Deficiency.^44^ The test was considered passed if all plates were read normally. All subjects passed the test.

##### Experiment 1

Data from one subject was excluded from analysis due to excessive noise in the EEG and data from an additional six subjects were excluded due to poor behavioral performance (see below for details). The analysis reported in the main text was performed on the data from the remaining 19 subjects (M_age_ = 28.2, SD_age_ = 5.2, range 21-43; 11 female, 8 male; 3 left-handed), who each performed 480 trials across 5 conditions (see below). According to a recent meta-analysis,^45^ this was sufficient to test our main hypothesis. We verified that our conclusions did not critically depend on the behavior-based participant exclusions (supplemental information).

##### Experiment 2

One subject was excluded from analysis due to not completing the full experimental session. Two subjects were excluded due to excessive noise in the EEG. Additionally, two more were excluded due to poor behavioral performance. The final sample size was 26 subjects (M_age_ = 28.2, SD_age_ = 5.1, range 20-39; 14 female, 12 male; 2 left-handed, 1 ambidextrous). As in Experiment 1, all subjects performed 480 trials across 5 conditions.

### Method details

#### Apparatus and stimuli

All tasks were completed on a Windows PC with a Dell E2422H monitor (24”, 1920 × 1080 pixels, 60 Hz refresh rate). The viewing distance was approximately 58 cm, maintained by positioning subjects’ chin on a chin rest. The tasks and stimuli were generated and displayed using MATLAB with the Psychophysics Toolbox Version 3 (PTB-3) extension.^46^^,^^47^^,^^48^ During the experiment, the subjects’ pupil size and eye movements were recorded using a Gazepoint GP3 eye tracker. Trials in which participants broke fixation by moving their gaze were automatically aborted, but the experimental session continued until they had successfully completed a total of 480 trials. A break in fixation was defined as the average gaze position straying more than 5.4° from the center of the screen.

Experiments 1 and 2 were structurally and visually identical, except for the format of the probe. The experimental session consisted of two parts, with a 5-10-minute rest period in between. Each part consisted of 240 trials, divided into 12 blocks of 20 trials each. Blocks were separated by 10-second breaks. Before the start of the experimental session, subjects completed a training block of 20 trials. EEG was not recorded during the training and the behavioral data were not included in the analysis.

On each trial subjects were presented with an array of four colored discs on a gray (RGB = 128 128 128) background. The colors of the discs were chosen from an isoluminant color wheel (CIE L∗a∗b∗ color space, [L = 70, a = −6, b = 14, radius = 49]), discretized to 180 colors and calibrated to the testing monitor. The colors were drawn from a uniform distribution on the color wheel but with the constraint that no two colors were separated by less than 30 degrees. In Experiment 1, subjects were asked to memorize two of the four presented colors, located in a particular hemifield. The relevant hemifield was randomized across trials and indicated by a pre-cue. In Experiment 2, subjects were instructed to ignore the colors.

#### Design and procedure

##### Trial structure

A trial started with a fixation period, during which a fixation point (two concentric circles: black, radius of 0.3°, and white, radius of 0.1°) was presented at the center of the screen. The subjects’ focus on the fixation point was monitored by the eye tracker to ensure 300 ms of uninterrupted fixation, meaning that moving their gaze away from the screen center extended the fixation period rather than aborting the trial. Breaking fixation at any later point, from the appearance of the cue and until the appearance of the probe, however, aborted the trial. A cue in the form of an arrow, indicating the to-be-attended side, was presented for 250 ms following the fixation period. Then, after a variable delay of 300-400 ms with only the fixation point visible, four colored discs (radius=1.3°) were presented for 500 ms, located at the corners of an invisible square centered on the fixation point. The distance between the fixation point and each stimulus was 8.1°. This was followed by a delay period of 1000 ms, during which only four identical black outlines of the discs remained on the screen, with their colors having disappeared at the start of the delay. Then, these outlines started moving along an invisible orbit centered on the fixation point, in identical direction and with identical speed, so that both the distances between the discs and the distance between the discs and the fixation point remained the same. The direction and angle of the movement was randomly selected from the range of [0°, 360°] in steps of 30°, meaning there were 13 possible angles in total. The movement was performed at a constant speed of 180°/s, meaning that its duration depended on the angle, with the shortest movement lasting 0 s (angle of 0°) and the longest movement lasting 2 s (angle of 360°). Another delay followed the movement, during which the discs’ outlines remained visible in their new locations. The duration of the delay was set to 3 s minus the duration of the movement, so that the total duration of movement and delay was always exactly 3 s, of which at least 1 s was delay. After the delay, in Experiment 1, the outlines of the discs disappeared and a memory probe – a color wheel – appeared at the new location of one of the previously cued discs. Subjects were asked to recall the color of that disc by picking it on the color wheel. Subjects had unlimited time to respond. After their response was submitted, they received feedback showing both the color they had chosen and the correct color for 500 ms, which concluded the trial. In Experiment 2, all outlines stayed on the screen, with one – the probe – changing its color to white. Subjects were asked to indicate whether the probe was one of the two discs they were tracking by pressing the “F” or “L” key. The keys were labeled with the words “YES” and “NO”, with the label assignment randomized between participants. The probe was a tracked disc in exactly half of the trials. After the response was submitted, the fixation point changed color to reflect whether it was correct (green) or incorrect (red). This feedback was shown for 500 ms. The intertrial interval was 1000 ms.

##### Conditions

We defined five experimental conditions, based on the final locations of the two cued discs: Condition 1 (0°, 30°), in which both discs remained in the original hemifield; Condition 2 (60°, 90°, 120°), in which one of the discs transferred to the other hemifield; Condition 3 (150°, 180°, 210°), in which both discs transferred to the other hemifield; Condition 4 (240°, 270°, 300°), in which both discs transferred to the other hemifield and then one of them returned to the original hemifield; and Condition 5 (330°, 360°), in which both discs transferred to the other hemifield and then returned to the original one.

##### Trial balancing

The attended hemifields were set to have equal numbers of trials (240) and within each attended hemifield, the numbers of trials per movement condition were likewise set to be equal (48). The numbers of trials per angle within each condition were balanced as well (24 or 16 for conditions with 2 or 3 angles, respectively). The sign of the angle, which determined whether the movement would be clockwise or counterclockwise, and the probed item were randomized across trials without enforcing equal numbers. Trial order was randomized, separately for each subject.

#### Participant exclusion based on poor behavioral accuracy

A pivotal aspect of our task design for Experiment 1 was that accurate performance was possible only if subjects mentally updated locations of stimuli held in WM as the disc outlines moved on the screen (see main text). Subjects with large response errors likely failed to perform this mental updating. Since our main interest was to study neural correlates of mental updating, data of such subjects were expected to be uninformative, essentially only adding noise. Therefore, we excluded subjects with unusually large errors from the main analyses. To this end, we imposed a performance criterion that excluded all subjects with an average response error of at least two standard deviations above the group mean, which was enforced iteratively until no subjects fulfilled this criterion. This resulted in a total of 6 exclusions (compare Figure 2A with Figure S10 to see the performance of the excluded subjects relative to the rest). To make sure that our main findings do not critically depend on this exclusion, we reran the analysis with these subjects included. As anticipated, the results were highly similar, albeit with reduced statistical strength due to a lowered signal-to-noise ratio in the EEG data (see Figures S9 and S11), suggesting that accurate performance was indeed pivotal. For Experiment 2, the same criterion was applied but only once, rather than iteratively, leading to 2 exclusions.

### Quantification and statistical analysis

#### Behavioral analysis

##### Experiment 1

For each participant we calculated the mean response error for each of the 13 motion angles. We calculated this error both with respect to the color of the target item and the color of the non-target (the cued but not probed) item. To assess how these two types of errors related to the motion angle, we used a Pearson correlation test, applied to participant-averaged errors (hence, 19 data points per correlation test). To estimate how the swap rate and memory precision related to the motion angle, we fitted an equal-precision model with non-target responses^20^ to the raw response data, separately for each participant. In this model, the response error on each trial was drawn from a Von Mises distribution with a concentration parameter *κ*. Moreover, there was a certain probability, *p*_swap_, that the response was based on the memory of the non-target item instead of the target. To investigate potential effects of motion angle *α* on memory precision and swap rate, we modelled both these variables as linear functions, through *κ*(*α*) = *a* + *bα* and *p*_swap_(*α*) = *c* + *dα,* where *a*, *b*, *c* and *d* were fitted as free parameters. The best-fitting parameters for each participant were found through maximum-likelihood estimation, that is, by finding the values of *a*, *b*, *c*, and *d* that maximized

$$\sum_{i=1}^{n_{\text{trials}}}\log\left[\left(1-p_{\text{swap}}\left(\alpha_{i}\right)\right)\text{VM}\left(\varepsilon_{\text{target},i},\kappa \left(\alpha_{i}\right)\right)+p_{\text{swap}}\left(\alpha_{i}\right)\text{VM}\left(\varepsilon_{\text{non}-\text{target},i},\kappa \left(\alpha_{i}\right)\right)\right]$$

where VM(*ε*, *κ*) is a zero-mean Von Mises distribution with concentration parameter *κ*, evaluated at *ε*; *ε*_target,*i*_ and *ε*_non-target,*i*_ denote the response errors relative to the target and non-target item, respectively, on trial *i*; *α*_*i*_ denotes the motion angle on trial *i.* The optimization was performed in Python, using the Bayesian Adaptive Direct Search (BADS) package.^49^

##### Experiment 2

Since the purpose of the task in Experiment 2 was to ensure that participants were actively tracking rather than passively viewing the movement, we did not perform any behavioral analyses apart from computing the performance accuracy defined as the ratio of correct responses to the total number of trials.

#### EEG recording

EEG was acquired at 2048 Hz from 64 active Ag/AgCl electrodes using BioSemi ActiveTwo system. The electrodes were placed on an elastic cap according to the International 10-20 system. Two additional electrodes were placed on the left and right mastoids. The vertical electrooculogram (VEOG), used to detect eye blinks, was recorded from electrodes located 2 cm above and below the right eye. The horizontal electrooculogram (HEOG), used to measure horizontal eye movements, was recorded from electrodes located 1 cm lateral to the external canthi.

#### EEG preprocessing

Preprocessing and analysis of the EEG data was carried out using MNE-Python.^43^ For the initial visual inspection, all electrodes were re-referenced offline to the average of the mastoids. If faulty electrodes were (manually) detected during or after the recording, they were interpolated using the MNE interpolate_bads() function with default parameters. This was done for 2 out of 190 (10 electrodes x 19 participants) electrodes kept in the main CDA analysis for Experiment 1, and for 9 out of 260 electrodes for Experiment 2. As excessive noise was discovered in the mastoid electrodes of several participants, EEG data were then re-referenced to the average of all 64 electrodes. The continuous data were bandpass-filtered using a finite impulse response (FIR) filter to 0.1-30 Hz and down-sampled to 256 Hz. A Picard^50^ Independent Component Analysis (ICA), as implemented in MNE, was then run on the filtered and down-sampled data and the obtained components were automatically tested (using MNE function find_bads_eog() that utilizes correlation with EOG channels) and manually inspected in order to detect and remove ocular artifacts (blinks and horizontal eye movements). The results of automatic detection aided the process, but the final decision was made based on manual inspection. EEG was epoched between -200 and 4500 ms time-locked to stimulus onset. Each epoch, therefore, included 200 ms of pre-stimulus fixation, 500 ms of stimulus presentation, 1000 ms of pre-movement delay, 0-2000 ms of stimuli movement, and 1000-3000 ms of post-movement delay.

Automatic trial rejection was used to remove trials contaminated with muscle artifacts, channels blocking, slow drifts, and step-like voltage changes in the electrodes used for the ERP analyses. The detection algorithms and rejection criteria were implemented as described in.^51^ The remaining trials were manually inspected to make sure no obvious artifacts had been missed. To aid reproducibility, manual rejection was substituted with an additional criterion of peak-to-peak amplitudes exceeding 150 μV, as the result of applying it was virtually identical. Subjects with fewer than 360 (75%) clean trials were excluded from analyses. Thus, for the remaining 19 subjects in Experiment 1, we rejected on average 1.6% (SD = 1.4%) of trials, resulting in an average of 472 trials per participant. For the 26 subjects in Experiment 2, an average of 2.4% (SD = 3.1%) trials were rejected, leaving an average of 468 clean trials per participant.

#### EEG analysis

##### CDA extraction

Artifact-free epochs were baseline-corrected using the 200 ms of pre-stimulus fixation as baseline and trial-averaged separately for left and right attended sides. All ERP analysis was restricted to the 10 posterior channels reported to show maximal CDA in memory tasks^52^: P5/P6, P7/P8, PO3/PO4, PO7/PO8, and O1/O2. The difference (CDA) waves were computed per participant, by subtracting their average activity recorded at ipsilateral sites from the average activity recorded at contralateral sites, with laterality defined relative to the attended (cued) side.

For statistical analysis, we measured CDA as the mean amplitude between 300 and 1000 ms post stimulus offset for pre-movement delay (analyzed in the supplemental information) and the mean amplitude between 300 and 1000 ms post movement end per condition in post-movement delay (main analysis). The movement end per condition was defined as the time point at which the longest movement included in the condition ended, e.g. 30° movement for Condition 1 and 210° movement for Condition 3.

##### Lateralized alpha power extraction

To obtain instantaneous alpha power, preprocessed EEG data were bandpass-filtered using a finite impulse response (FIR) filter to 8-12 Hz. A Hilbert transform was applied to the filtered data and the instantaneous power was calculated as the square of the absolute value of the complex signal. The continuous data was then epoched the same way as in the CDA analyses, but no baseline correction was applied. Lateralized alpha power for each condition was computed as the difference between contralateral and ipsilateral average power over the 10 posterior channels used to compute CDA. The time windows used for statistical analyses of mean lateralized alpha power were also consisted with the CDA analyses.

##### Frequentist t-tests

To test the predictions of our main model against the data, we performed Student’s t-tests on the time-averaged CDA measures. The null hypothesis was specified as H_0_: *μ*=0. The model predicted H_0_ to be true in Conditions 2 and 4 and false in Conditions 1, 3, and 5. Since the model predicted a specific effect direction in each of the latter three conditions, we used one-tailed tests for those conditions. For the two conditions where the null hypothesis was predicted to be true, we used a two-tailed test.

##### Bayesian t-tests

A limitation of frequentist tests is that they can only provide evidence *against* the null hypothesis. A high *p*-value means that the data do not provide evidence against the null hypothesis, but it would be a statistical fallacy to interpret such a finding as evidence *in favor* of the null.^53^ Therefore, we also analyzed the data using Bayesian t-tests. This type of test quantifies evidence by means of a Bayes Factor (BF), expressing how strongly the data support one hypothesis relative to another hypothesis. BFs above 1 provide support for a given hypothesis, below 1 evidence against. We use the interpretation scale from^54^ to map Bayes factors to verbal labels such as ‘weak’ and ‘strong’ evidence. An advantage of BFs is that they can quantify evidence both for and against any well-defined hypotheses, including the null.^55^ In the Bayesian evaluation of the model predictions, we computed for each condition the BF for the hypothesis following from our main model, denoted H_M_, relative to an alternative hypothesis H_A_, as listed in *Overview of hypotheses tested in the Bayesian t-tests*. Note that in Condition 1 we compared the model prediction, H_M_: *μ*<0, only against “*μ*=0”, not against “*μ*>0”. Our motivation for this was that published literature on CDA consistently reports the amplitude to be negative or non-existent and rarely or never as positive. Therefore, “*μ*>0” has near-zero prior plausibility and should, therefore, not be included when doing Bayesian testing (including it would have increased the evidence for our model, so the reported value can be considered as a conservative estimate). For the other conditions we had no strong prior expectations, because there is no prior literature on the effect of mental updating on CDA. Therefore, we specified the alternative hypothesis in those conditions as the complement of the model’s hypothesis (e.g., when the model predicts “>0”, the alternative would be defined as “≤0”). We computed BFs using the Bayesian t-test in JASP,^56^ which allows to test four different types of hypotheses (*μ*<0, *μ*=0, *μ*>0, *μ*≠0) against each other. A limitation is that it does not allow to directly test the “compound” alternative hypotheses that we specified for Conditions 3 and 5. Instead, we computed these BFs as the average BF obtained from testing H_M_ separately against each component in the compound hypothesis. Hence, in Condition 3, we took the average of the BF obtained from testing H_M_: μ>0 against H_A_: μ=0 and the BF obtained from testing H_M_: μ>0 against H_A_: μ<0. The latter BF (for H_M_: μ>0 against H_A_: μ<0) could also not be computed directly in JASP. Instead, we computed it as the ratio of BF_+0_ and BF_−0_: ${\text{BF}}_{+-}=\frac{p\left(D|\mu >0\right)}{p\left(D|\mu <0\right)}=\frac{\left(\frac{p\left(D|\mu >0\right)}{p\left(D|\mu =0\right)}\right)}{\left(\frac{p\left(D|\mu <0\right)}{p\left(D|\mu =0\right)}\right)}=\frac{{\text{BF}}_{+0}}{{\text{BF}}_{-0}}$. The BF for Condition 5 was computed in a similar way. In all Bayesian t-tests, the default prior was used (a Cauchy distribution with a scale parameter of .707).

**Table:** 

Condition	H_M_	H_A_	Computation of BF_MA_
1	*μ* < 0	*μ* = 0	BF_MA_ = BF_-0_
2	*μ* = 0	*μ* ≠ 0	BF_MA_ = BF_01_
3	*μ* > 0	*μ* ≤ 0	${\text{BF}}_{\text{MA}}=\frac{1}{2}{\text{BF}}_{+0}+\frac{1}{2}\frac{{\text{BF}}_{+0}}{{\text{BF}}_{-0}}$
4	*μ* = 0	*μ* ≠ 0	BF_MA_ = BF_01_
5	*μ* < 0	*μ* ≥ 0	${\text{BF}}_{\text{MA}}=\frac{1}{2}{\text{BF}}_{-0}+\frac{1}{2}\frac{{\text{BF}}_{-0}}{{\text{BF}}_{+0}}$

#### Overview of hypotheses tested in the Bayesian t-tests

For each condition, we calculated a Bayes Factor BF_MA_, quantifying the evidence in favor of the hypothesis H_M_ relative to H_A_. Bayes Factors larger than 1 indicate support for the model prediction, while Bayes Factors smaller than 1 indicate evidence against the model prediction.

##### Cluster-based permutation tests

For the additional analyses of the combined Conditions 3-5, we conducted one-tailed cluster-based permutation tests as implemented in the MNE function mne.stats.permutation_cluster_1samp_test. The clusters were defined as adjacent data points (in time) that exceeded a t-statistic threshold calculated for *p* = .05 and the number of participants in each experiment. Cluster level statistics were calculated as the sum of the t-values within a cluster. The tests were run with 10,000 permutations.

##### Robustness checks

To verify that our statistical results did not critically depend on the specific time windows used in the CDA calculations, we also ran the analyses with time windows locked to the movement end for each individual motion angle (resulting in 13 separate time windows instead of 5) and with a single window that was time-locked to the end of post-movement delay. The results (see Figure S3 and Supplemental text) were consistent with the results reported in the main text.

#### Bayesian mixed-effects modeling

To evaluate the three competing hypotheses about CDA dynamics on all 5 conditions at once, we fitted Bayesian mixed-effects models to post-movement CDA amplitudes (300 – 1000 ms). Analyses were performed in Python using the *bambi* package with posterior sampling conducted via Markov Chain Monte Carlo (4 chains, 4000 draws after 2000 warm-up, target acceptance 0.95). CDA amplitudes were expressed in microvolts. All models included subject-level random intercepts; when condition-specific effects were estimated, corresponding subject-level random slopes were included.

The three models corresponded to the theoretical predictions tested in the study. In Model 1 (hemispheric transfer), the intercept was omitted and predictors were coded −1 for conditions predicted to show negative CDA effects (Conditions 1 and 5) and +1 for the condition predicted to show a positive effect (Condition 3), while Conditions 2 and 4 were fixed to zero. In Model 2 (uniform CDA), a single intercept was fitted representing the common mean CDA amplitude across all five conditions and constrained to be negative. In Model 3, a negative effect was estimated for Condition 1, while the remaining conditions were fixed to zero.

Directional constraints in Models 1 and 3 were implemented using Half-Normal priors on coefficient magnitudes (*σ* = 1.5 μV), such that the sign of the predicted effect was determined by the ±1 predictor coding. In Model 2, the intercept was assigned a truncated normal prior constrained to negative values. Model fits were compared using leave-one-out cross-validation (LOO) with stacking weights, which estimate the relative predictive contribution of each model in a model ensemble.^57^ Posterior estimates are reported as posterior means and 95% highest density intervals (HDIs).

#### Between-experiment comparison

To directly compare the CDA patterns in Experiments 1 and 2, we fitted an additional Bayesian hierarchical model to the post-movement CDA amplitudes (300–1000 ms) across all five conditions:

CDA∼0+experiment:condition+(0+condition|subject)

This model estimated CDA amplitudes for each experiment × condition combination while allowing subject-specific deviations across conditions. Posterior samples were used to compute contrasts quantifying (i) the difference between experiments in Condition 3 and (ii) the magnitude of the CDA reversal in Condition 3 relative to Conditions 1 and 5 (as the difference between CDA in Condition 3 and the average CDA of Conditions 1 and 5).

#### Gaze bias analyses

To evaluate the possible effects of systematic eye movements on the CDA, we analyzed average horizontal gaze bias in the same post-movement time window that was used in the CDA analyses. We defined horizontal gaze bias at time *t* as the distance, on the x-axis, between point of gaze (POG, averaged between left and right eye) at time *t* and the average POG during fixation. To account for possible drift over the course of the experimental session, we computed trial-specific average POG during the 300 ms fixation at the start of each trial, used it to calculate the horizontal gaze bias at each timepoint of the post-movement time window, and averaged the bias within the time window, resulting in a single bias value per trial. We then flipped the sign on left-cued trials so that the bias in the direction of the cue was always positive. Finally, we averaged the horizontal gaze bias within each condition, resulting in one value per condition per participant. These average gaze bias values were used for statistical analyses (see Figures S7 and S8 and Supplemental text).

#### Source estimation

As a supplemental exploratory analysis, we investigated source-level activity to test whether the observed post-movement CDA reflects a relocation of activity across cortex rather than, for example, changes in dipole orientation. Using a linearly constrained minimum variance (LCMV) beamformer,^58^ as implemented in MNE-Python, with a template MRI (fsaverage), a cortically constrained source space ('fsaverage‘-ico-5-src'), and a standard electrode montage (‘standard_1005’), we obtained source estimates of the evoked activity for the post-movement time windows that were used in the CDA analyses. The noise covariance was computed using the 200 ms pre-stimulus baseline and the data covariance was computed using the entire post-movement delay (from 300 ms after movement end of Condition 1 until the onset of the probe). Both the noise covariance and the data covariance were computed using all the clean trials and the resulting LCMV filter was applied to all conditions. The rest of the parameters were kept at default MNE-Python values (version 1.8.0). The source estimates were computed separately for each condition and cue, the hemispheres of the right-cued source estimates were flipped so that for both cues the left hemisphere represented the ipsilateral and the right hemisphere represented the contralateral side, and then the two were averaged to obtain one source estimate per condition per participant. For both visualization and statistical analyses (Figure S4 and Supplemental text), we used a broad posterior region of interest (ROI) consisting of the following parieto-occipital labels from the Desikan-Killiany (DK) atlas^59^: lateraloccipital, cuneus, pericalcarine, lingual, superiorparietal, inferiorparietal, precuneus. Statistical analyses were applied to the difference between mean ROI activity over the contralateral and ipsilateral hemispheres.

## References

[1] D’Esposito M., Postle B.R. (2015). The cognitive neuroscience of working memory. Annu. Rev. Psychol..

[2] Baddeley A. (1992). Working memory. Science.

[3] Stokes M.G. (2015). “Activity-silent” working memory in prefrontal cortex: a dynamic coding framework. Trends Cogn. Sci..

[4] Goldman-Rakic P.S. (1995). Cellular basis of working memory. Neuron.

[5] Lundqvist M., Rose J., Herman P., Brincat S.L., Buschman T.J., Miller E.K. (2016). Gamma and beta bursts underlie working memory. Neuron.

[6] Rose N.S., LaRocque J.J., Riggall A.C., Gosseries O., Starrett M.J., Meyering E.E., Postle B.R. (2016). Reactivation of latent working memories with transcranial magnetic stimulation. Science.

[7] Christophel T.B., Iamshchinina P., Yan C., Allefeld C., Haynes J.-D. (2018). Cortical specialization for attended versus unattended working memory. Nat. Neurosci..

[8] Sprague T.C., Ester E.F., Serences J.T. (2016). Restoring latent visual working memory representations in human cortex. Neuron.

[9] Panichello M.F., Buschman T.J. (2021). Shared mechanisms underlie the control of working memory and attention. Nature.

[10] Sauseng P., Griesmayr B., Freunberger R., Klimesch W. (2010). Control mechanisms in working memory: a possible function of EEG theta oscillations. Neurosci. Biobehav. Rev..

[11] Yu Q., Teng C., Postle B.R. (2020). Different states of priority recruit different neural representations in visual working memory. PLoS Biol..

[12] Brincat S.L., Donoghue J.A., Mahnke M.K., Kornblith S., Lundqvist M., Miller E.K. (2021). Interhemispheric transfer of working memories. Neuron.

[13] Draschkow D., Nobre A.C., van Ede F. (2022). Multiple spatial frames for immersive working memory. Nat. Hum. Behav..

[14] Vogel E.K., McCollough A.W., Machizawa M.G. (2005). Neural measures reveal individual differences in controlling access to working memory. Nature.

[15] Vogel E.K., Machizawa M.G. (2004). Neural activity predicts individual differences in visual working memory capacity. Nature.

[16] Kuo B.-C., Stokes M.G., Nobre A.C. (2012). Attention modulates maintenance of representations in visual short-term memory. J. Cogn. Neurosci..

[17] Feldmann-Wüstefeld T., Vogel E.K., Awh E. (2018). Contralateral delay activity indexes working memory storage, not the current focus of spatial attention. J. Cogn. Neurosci..

[18] Wilken P., Ma W.J. (2004). A detection theory account of change detection. J. Vis..

[19] Bays P.M., Catalao R.F.G., Husain M. (2009). The precision of visual working memory is set by allocation of a shared resource. J. Vis..

[20] van den Berg R., Awh E., Ma W.J. (2014). Factorial comparison of working memory models. Psychol. Rev..

[21] Luria R., Balaban H., Awh E., Vogel E.K. (2016). The contralateral delay activity as a neural measure of visual working memory. Neurosci. Biobehav. Rev..

[22] Günseli E., Fahrenfort J.J., van Moorselaar D., Daoultzis K.C., Meeter M., Olivers C.N.L. (2019). EEG dynamics reveal a dissociation between storage and selective attention within working memory. Sci. Rep..

[23] Drew T., Horowitz T.S., Wolfe J.M., Vogel E.K. (2011). Delineating the neural signatures of tracking spatial position and working memory during attentive tracking. J. Neurosci..

[24] Drew T., Mance I., Horowitz T.S., Wolfe J.M., Vogel E.K. (2014). A soft handoff of attention between cerebral hemispheres. Curr. Biol..

[25] Berggren N., Eimer M. (2016). Does Contralateral Delay Activity Reflect Working Memory Storage or the Current Focus of Spatial Attention within Visual Working Memory?. J. Cogn. Neurosci..

[26] Hakim N., Adam K.C.S., Gunseli E., Awh E., Vogel E.K. (2019). Dissecting the Neural Focus of Attention Reveals Distinct Processes for Spatial Attention and Object-Based Storage in Visual Working Memory. Psychol. Sci..

[27] Hakim N., Feldmann-Wüstefeld T., Awh E., Vogel E.K. (2020). Perturbing Neural Representations of Working Memory with Task-irrelevant Interruption. J. Cogn. Neurosci..

[28] Grubert A., Eimer M. (2015). Does visual working memory represent the predicted locations of future target objects? An event-related brain potential study. Brain Res..

[29] Wang S., Rajsic J., Woodman G.F. (2019). The Contralateral Delay Activity Tracks the Sequential Loading of Objects into Visual Working Memory, Unlike Lateralized Alpha Oscillations. J. Cogn. Neurosci..

[30] Luria R., Vogel E.K. (2014). Come together, right now: dynamic overwriting of an object’s history through common fate. J. Cogn. Neurosci..

[31] Balaban H., Luria R. (2015). The number of objects determines visual working memory capacity allocation for complex items. Neuroimage.

[32] Balaban H., Luria R. (2017). Neural and behavioral evidence for an online resetting process in visual working memory. J. Neurosci..

[33] Styrkowiec P.P., Ngiam W.X.Q., Epstein W., Gneezy R., Jones H.M., Awh E., Vogel E.K. (2026). Item-based Parsing of Dynamic Scenes in a Combined Attentional Tracking and Working Memory Task. J. Cogn. Neurosci..

[34] Wang Y., Lin X., Ye C., Parviainen T., Liu Q. (2025). Contralateral Delay Activity Tracks Storage Load in Visual Working Memory: Evidence from the Multiple Object Tracking Task. J. Cogn. Neurosci..

[35] Awh E., Vogel E.K. (2025). Working memory needs pointers. Trends Cogn. Sci..

[36] Emrich S.M., Salahub C., Katus T. (2022). Sensory Delay Activity: More than an Electrophysiological Index of Working Memory Load. J. Cogn. Neurosci..

[37] Gao Z., Li J., Liang J., Chen H., Yin J., Shen M. (2009). Storing fine detailed information in visual working memory--evidence from event-related potentials. J. Vis..

[38] Luria R., Sessa P., Gotler A., Jolicoeur P., Dell’Acqua R. (2010). Visual short-term memory capacity for simple and complex objects. J. Cogn. Neurosci..

[39] Asp I.E., Störmer V.S., Brady T.F. (2021). Greater visual working memory capacity for visually matched stimuli when they are perceived as meaningful. J. Cogn. Neurosci..

[40] Thibeault A.M.L., Stojanoski B., Emrich S.M. (2024). Investigating the effects of perceptual complexity versus conceptual meaning on the object benefit in visual working memory. Cogn. Affect. Behav. Neurosci..

[41] Xie W., Zhang W. (2018). Familiarity Speeds Up Visual Short-term Memory Consolidation: Electrophysiological Evidence from Contralateral Delay Activities. J. Cogn. Neurosci..

[42] Kandemir G., Wilhelm S.A., Axmacher N., Akyürek E.G. (2024). Maintenance of color memoranda in activity-quiescent working memory states: Evidence from impulse perturbation. iScience.

[43] Gramfort A., Luessi M., Larson E., Engemann D.A., Strohmeier D., Brodbeck C., Goj R., Jas M., Brooks T., Parkkonen L., Hämäläinen M. (2013). MEG and EEG data analysis with MNE-Python. Front. Neurosci..

[44] Ishihara S. (1960). Tests for Colour-Blindness.

[45] Ngiam W.X.Q., Adam K.C.S., Quirk C., Vogel E.K., Awh E. (2021). Estimating the statistical power to detect set-size effects in contralateral delay activity. Psychophysiology.

[46] Brainard D.H. (1997). The Psychophysics Toolbox. Spat. Vis..

[47] Pelli D.G. (1997). The VideoToolbox software for visual psychophysics: transforming numbers into movies. Spat. Vis.

[48] Kleiner M., Brainard D., Pelli D., Ingling A., Murray R., Broussard C. (2007). What’s new in psychtoolbox-3. Perception.

[49] Acerbi L., Ma W.J. (2017). Practical Bayesian Optimization for Model Fitting with Bayesian Adaptive Direct Search. Adv. Neural Inf. Process. Syst..

[50] Ablin P., Cardoso J.-F., Gramfort A. (2018). Faster independent component analysis by preconditioning with hessian approximations. IEEE Trans. Signal Process..

[51] Hakim N., Feldmann-Wüstefeld T., Awh E., Vogel E.K. (2021). Controlling the flow of distracting information in working memory. Cereb. Cortex.

[52] Emrich S.M., Al-Aidroos N., Pratt J., Ferber S. (2009). Visual search elicits the electrophysiological marker of visual working memory. PLoS One.

[53] Goodman S. (2008). A dirty dozen: twelve p-value misconceptions. Semin. Hematol..

[54] Wagenmakers E.-J., Love J., Marsman M., Jamil T., Ly A., Verhagen J., Selker R., Gronau Q.F., Dropmann D., Boutin B. (2018). Bayesian inference for psychology. Part II: Example applications with JASP. Psychon. Bull. Rev..

[55] Wagenmakers E.-J., Marsman M., Jamil T., Ly A., Verhagen J., Love J., Selker R., Gronau Q.F., Šmíra M., Epskamp S. (2018). Bayesian inference for psychology. Part I: Theoretical advantages and practical ramifications. Psychon. Bull. Rev..

[56] JASP Team (2024).

[57] Yao Y., Vehtari A., Simpson D., Gelman A. (2018). Using stacking to average bayesian predictive distributions. Bayesian Anal..

[58] Van Veen B.D., van Drongelen W., Yuchtman M., Suzuki A. (1997). Localization of brain electrical activity via linearly constrained minimum variance spatial filtering. IEEE Trans. Biomed. Eng..

[59] Desikan R.S., Ségonne F., Fischl B., Quinn B.T., Dickerson B.C., Blacker D., Buckner R.L., Dale A.M., Maguire R.P., Hyman B.T. (2006). An automated labeling system for subdividing the human cerebral cortex on MRI scans into gyral based regions of interest. Neuroimage.

