# Supplementary material for: Contralateral delay activity during dynamic spatial updates in working memory
Source: iScience. 2026 Oct 19;29(11):117916. doi: 10.1016/j.isci.2026.117916 (PMC13697815; doi:10.1016/j.isci.2026.117916)
Supplement: Supplementary file 1 [file mmc1.pdf]

iScience, Volume *29*

Supplemental information

Contralateral delay activity during dynamic spatial updates in working memory

Charles Chernik, Ronald van den Berg, and Mikael Lundqvist

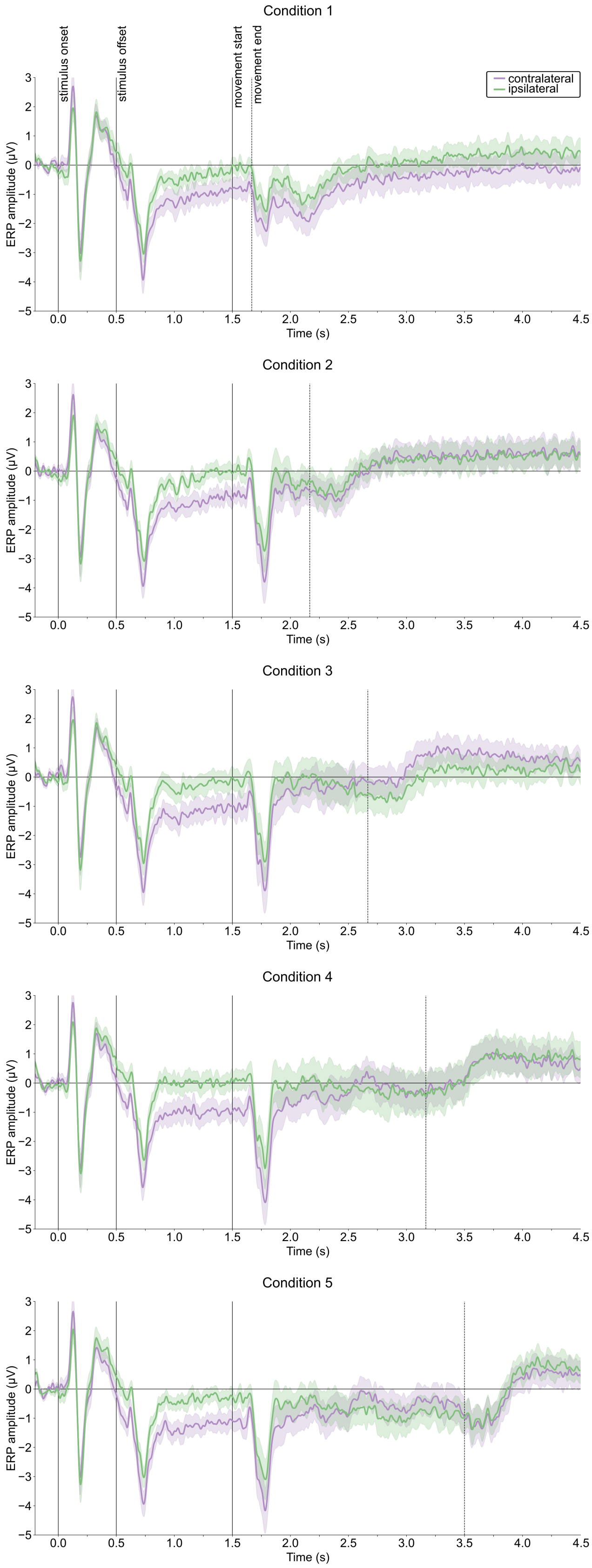

Figure S1. Average ERP waveforms from contralateral and ipsilateral electrodes in Experiment 1. Related to Figure 3.

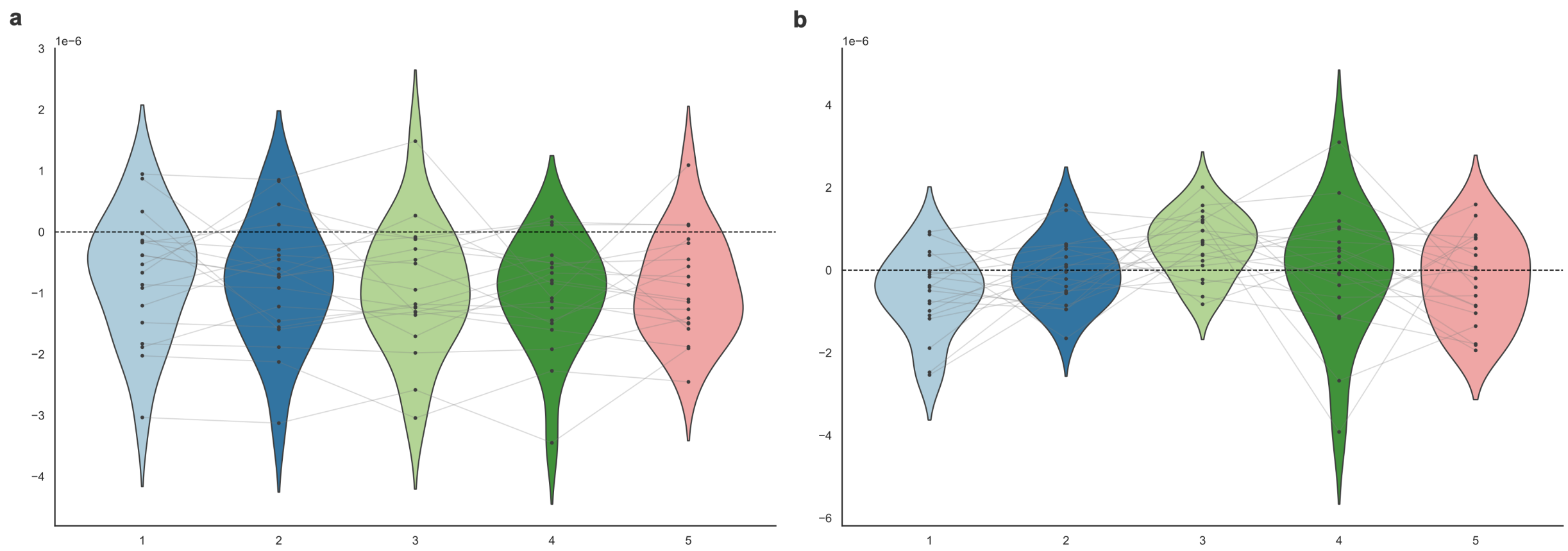

Figure S2. Distribution of average CDA amplitudes per condition in Experiment 1. **a.** Before the movement start (300-1000 ms post stimulus offset). **b.** After the movement (same time window as in Figure 3c). Black dots represent average CDA of individual participants, with light gray lines connecting data from the same participant across conditions. Related to Figure 3.

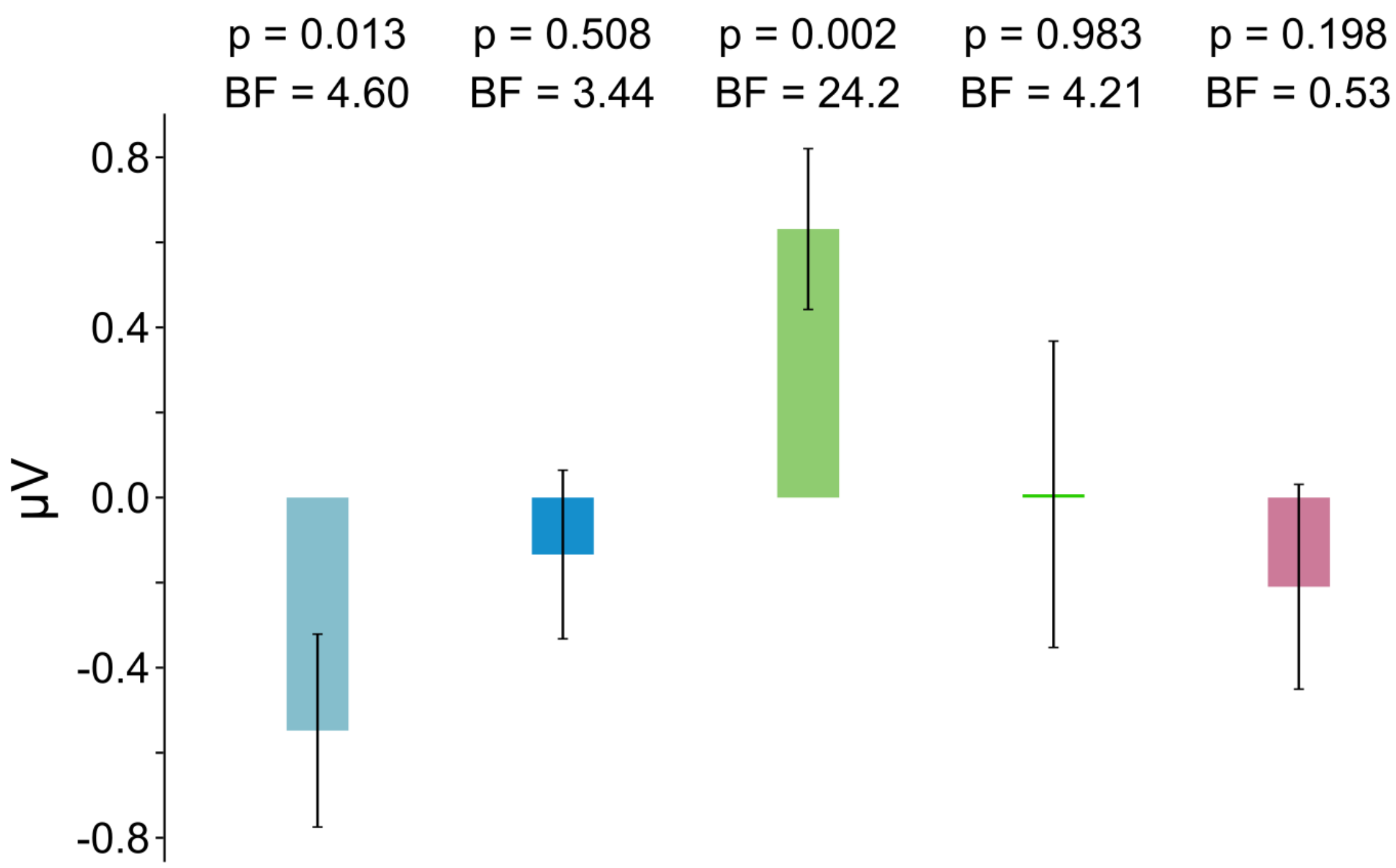

Figure S3. Same as Figure 3c, but with average CDA amplitude obtained from time windows locked to 300-1000 ms post movement end rather than time locked to condition end. Error bars denote SEM. The p-values were obtained from t-tests against the null hypothesis of CDA being 0. The BFs were obtained from Bayesian t-tests and quantify the evidence in favor of the predictions made by Model 1 (see Methods). Related to Figure 3.

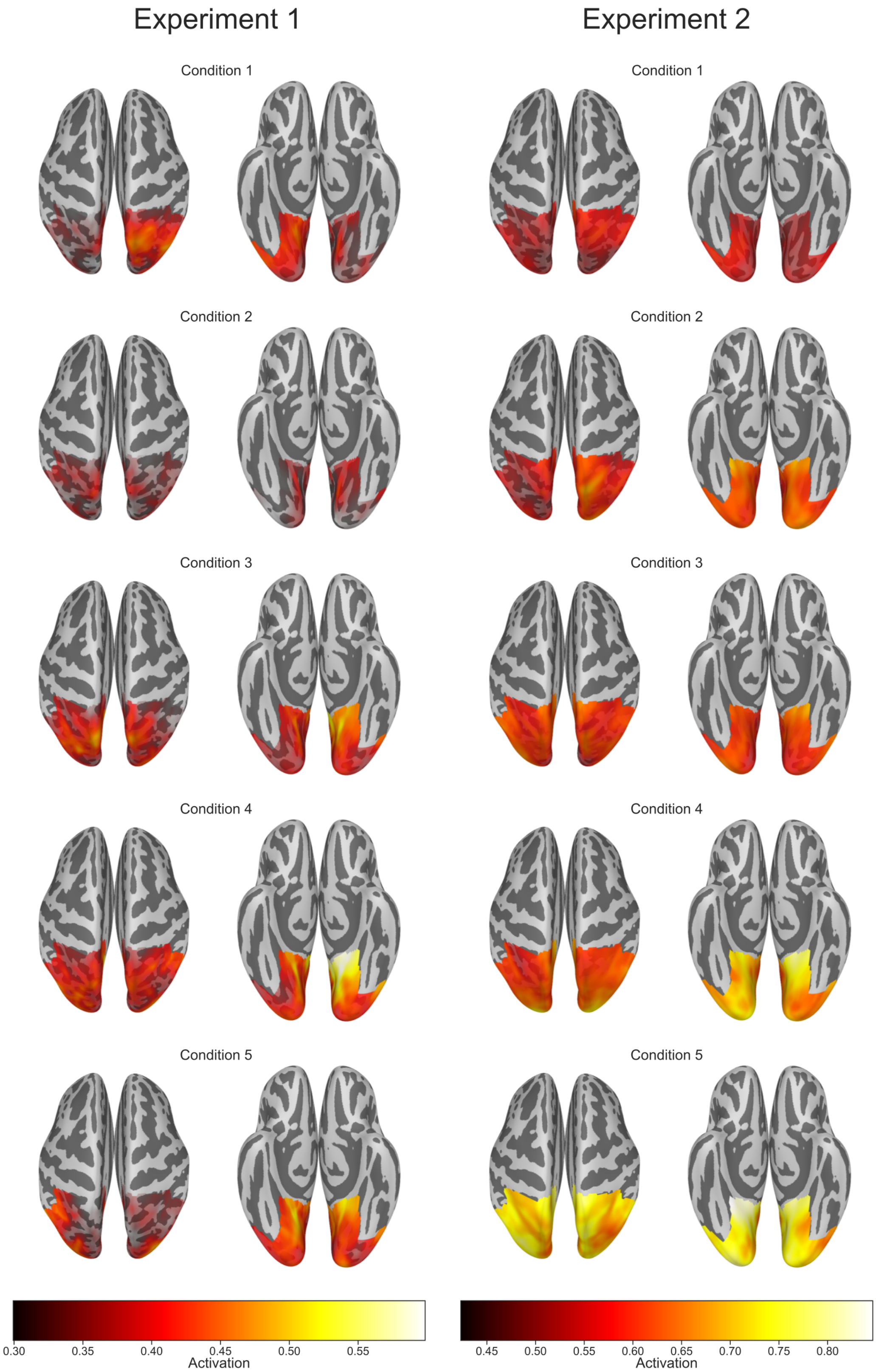

Figure S4. Grand-averaged source estimates over the posterior ROI for Experiments 1 and 2. Left hemisphere represents the ipsilateral and right hemisphere the contralateral side relative to cue direction. Related to Figure 3 and STAR Methods.

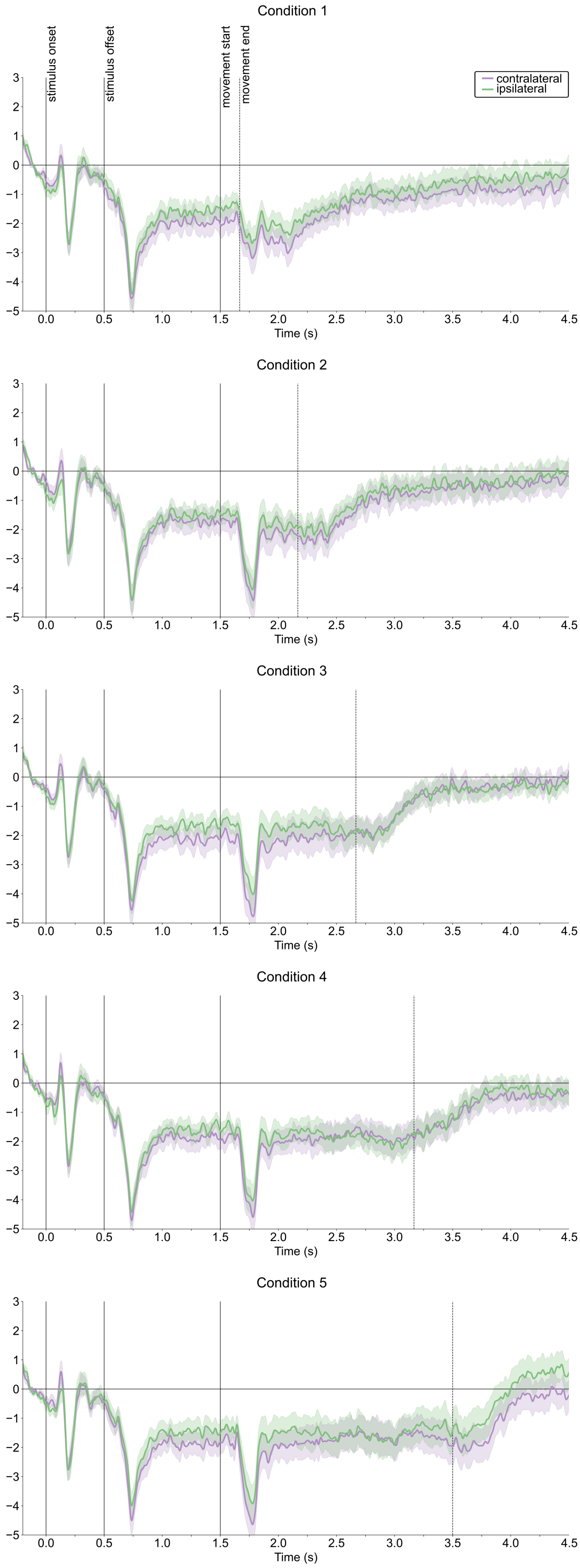

Figure S5. Average ERP waveforms from contralateral and ipsilateral electrodes in Experiment 2. Related to Figure 3.

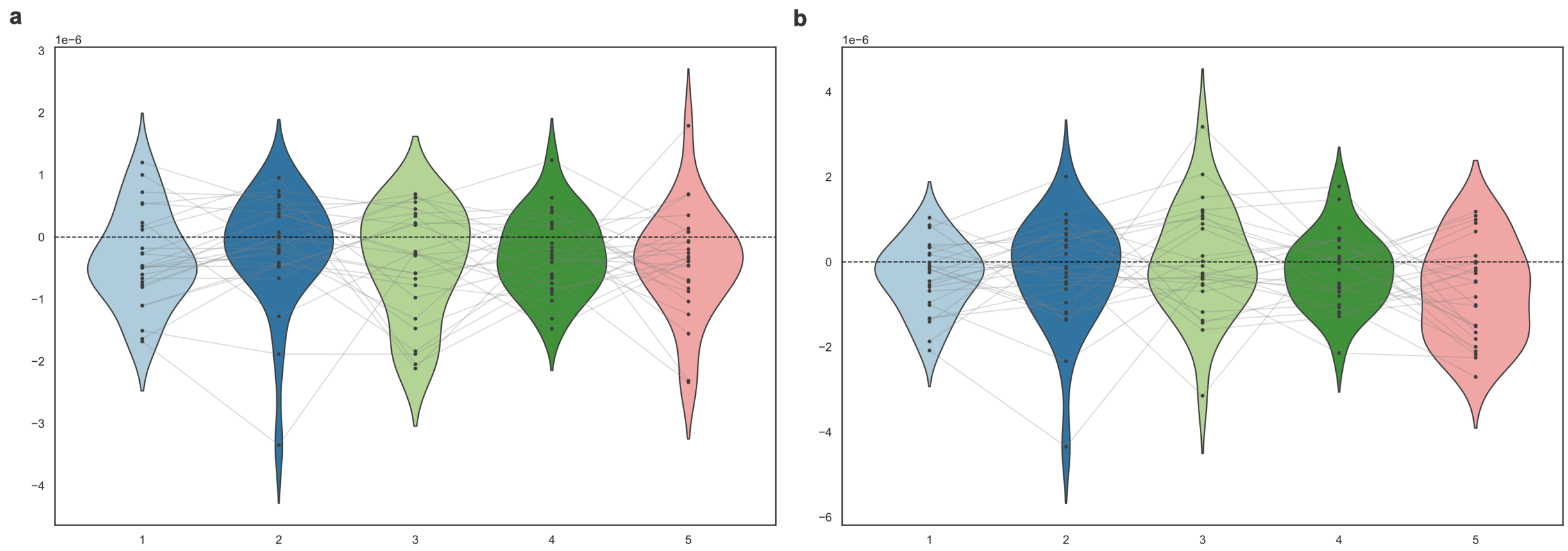

Figure S6. Distribution of average CDA amplitudes per condition in Experiment 2. **a.** Before the movement start (300-1000 ms post stimulus offset). **b.** After the movement (same time window as in Figure 3e). Black dots represent average CDA of individual participants, with light gray lines connecting data from the same participant across conditions. Related to Figure 3.

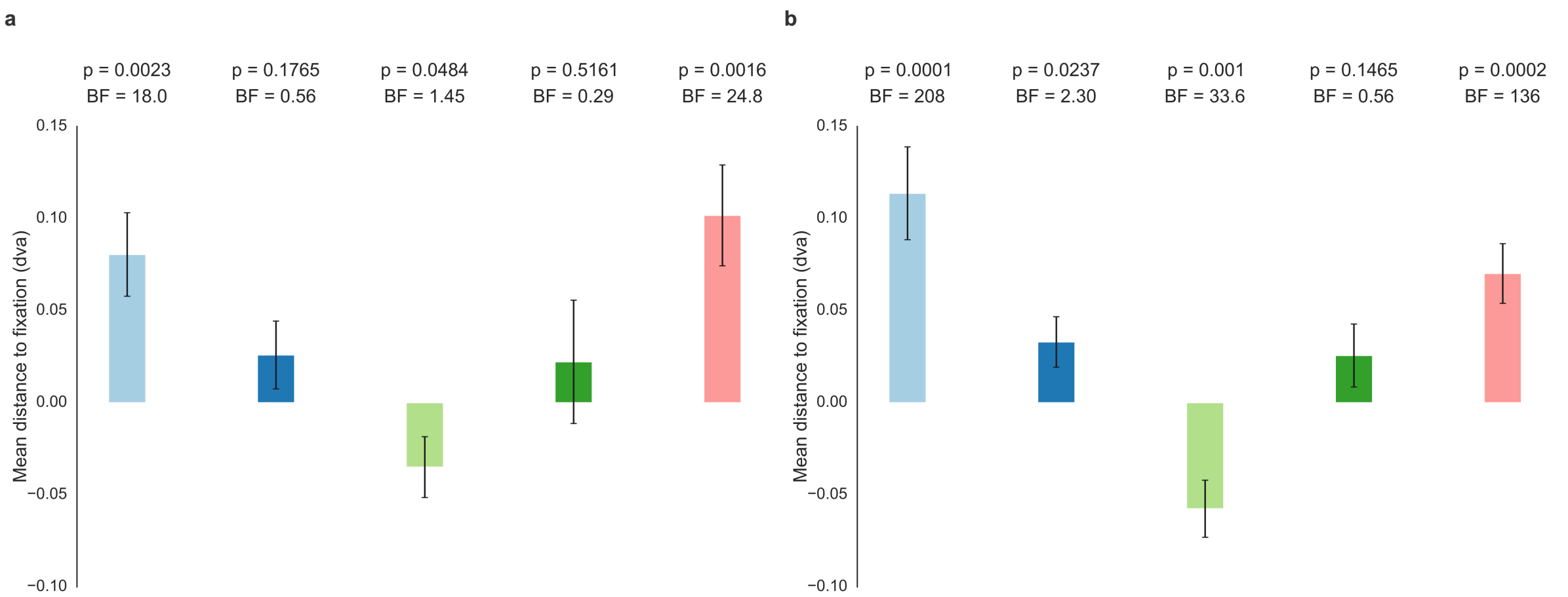

Figure S7. Average horizontal gaze bias during the post-movement delay for Experiment 1 (a) and Experiment 2 (b). Error bars denote SEM. Positive values correspond to bias towards the initially cued side. The p-values were obtained from two-tailed t-tests against the null hypothesis of gaze bias being 0. The BFs were obtained from Bayesian t-tests and quantify the evidence in favor of presence of gaze bias (two-sided). Related to Figure 3 and STAR Methods.

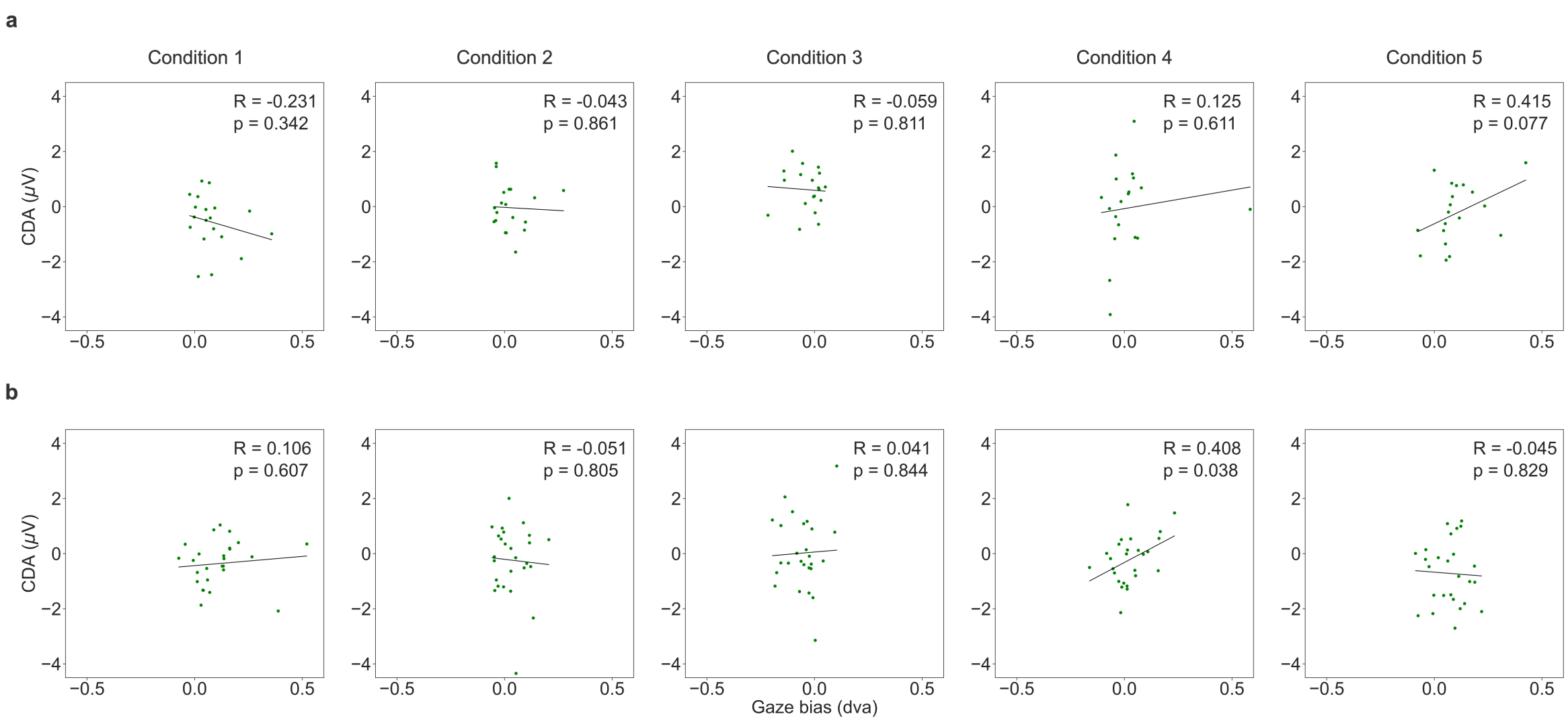

Figure S8. Linear regression between gaze bias and CDA post movement for each condition in Experiment 1 (a) and Experiment 2 (b). Related to Figure 3 and STAR Methods.

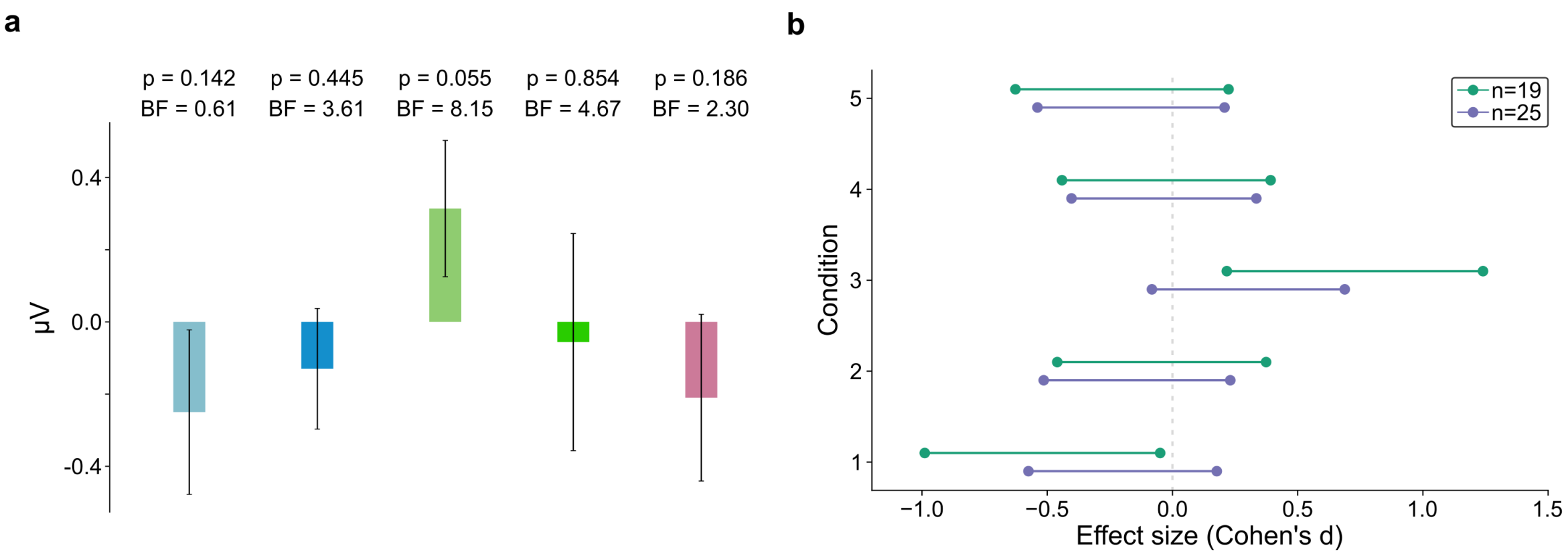

Figure S9. a. Same as Figure 3c, but for 25 subjects (including the ones excluded from main analysis based on behavior). Error bars denote SEM. **b.** Bayesian 95% credible intervals for CDA amplitude, split by condition and presented separately for the cases with behavior-based exclusions (n=19, as in the main text; green) and without such exclusions (n=25; purple). The CDAs were taken from the time periods indicated in Figure 3b. Related to Figure 3.

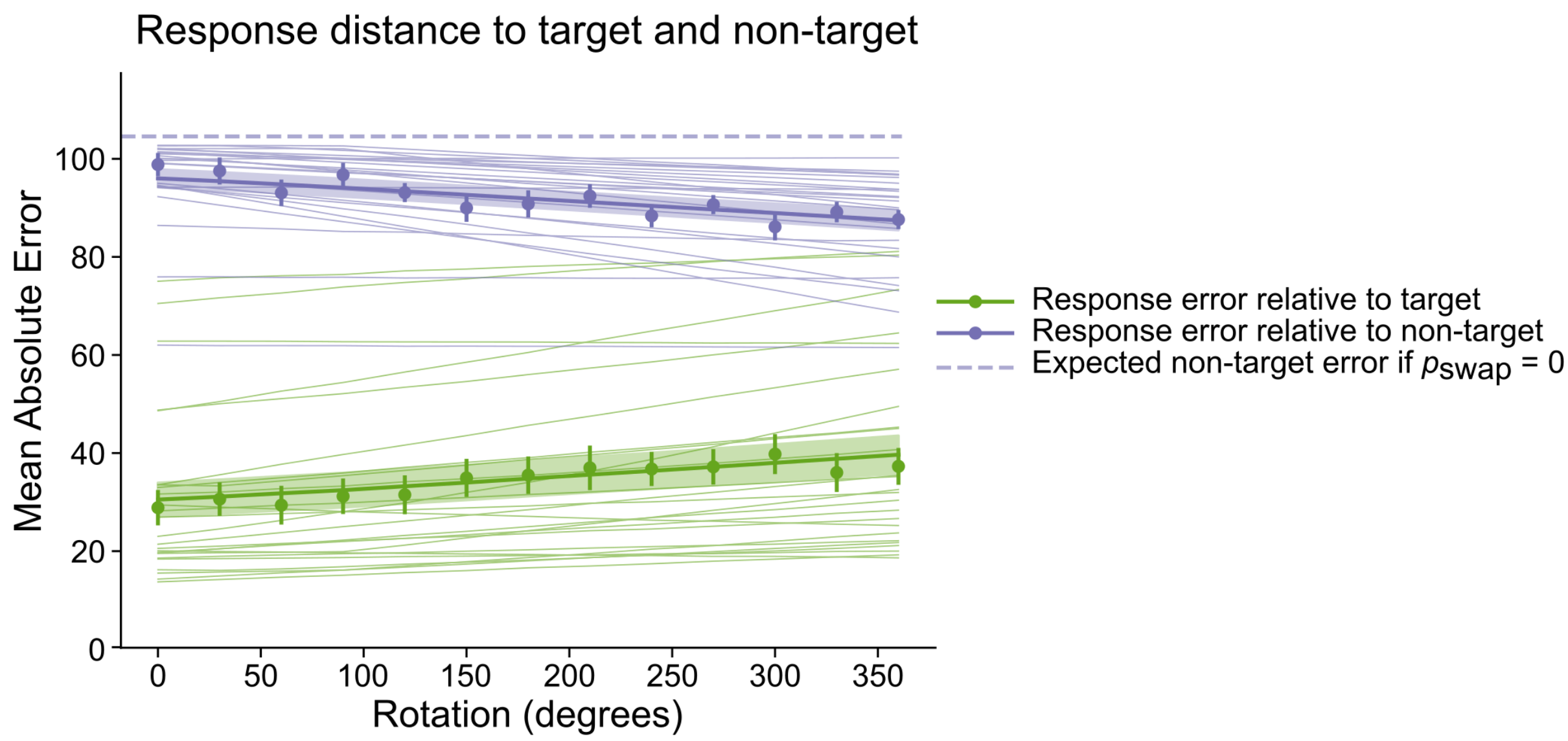

Figure S10. Same as Figure 2a, but for 25 subjects (including the ones excluded from main analysis based on behavior). Shaded areas and error bars represent one SEM. Related to Figure 2.

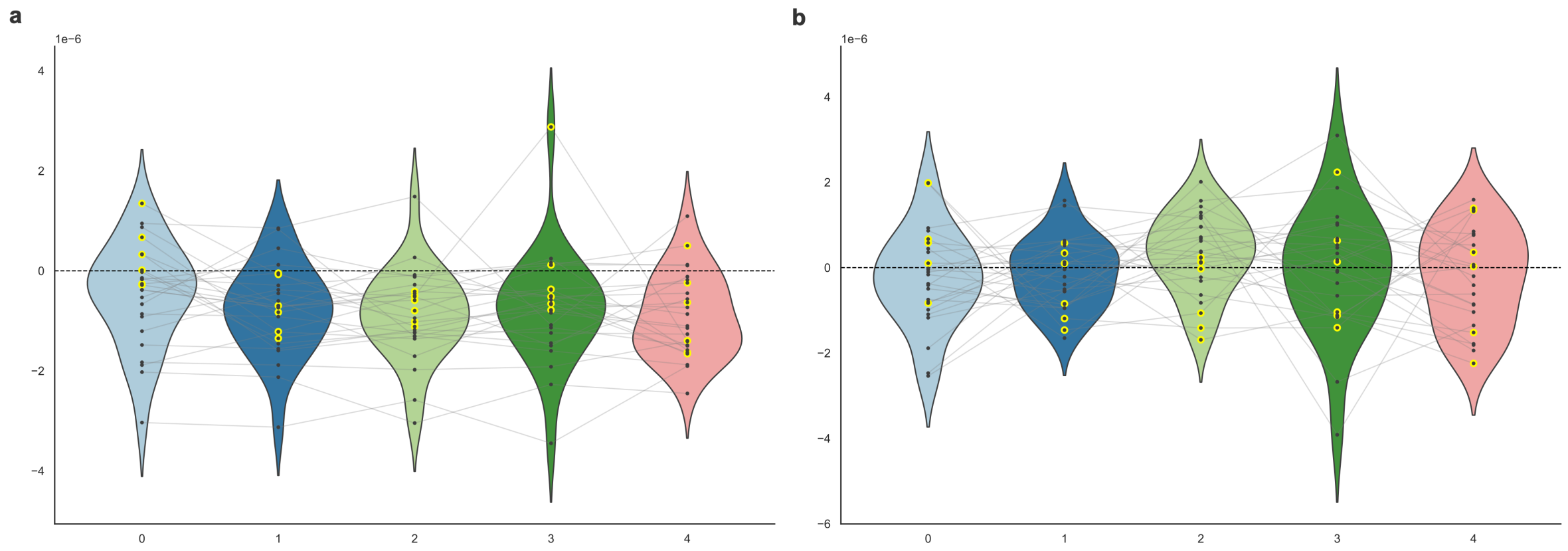

Figure S11. Same as Figure S2, but for 25 subjects (including the ones excluded from main analysis based on behavior, highlighted in yellow). Related to Figure 3 and STAR Methods.

Supplemental text:

We additionally performed Bayesian t-tests to test Model 1 in the same way as presented in Figures 3c and 3e, but instead with CDA windows time-locked to the end of post-movement delay (last 700 ms) rather than to the movement end. For Experiment 1, the BFs for this time window per condition were: condition 1: BF = *3.30* ("<0" vs "=0"); condition 2: BF = *3.97* ("=0" vs "≠0"); condition 3: BF = 4.67 (">0" vs "≤0"); condition 4: BF = *3.83* ("=0" vs "≠0"); condition 5: BF = 0.60 ("<0" vs "≥0"). For Experiment 2: condition 1: BF = 1.76; condition 2: BF = 4.06; condition 3: BF = 0.29; condition 4: BF = 2.56; condition 5: BF = 20.2.

To test for a source-level reversal of the hemispheric activity profile, we performed the same profile modulation analysis as we applied to the CDA data, comparing source-level activity over the posterior ROI in Condition 3 to Conditions 1 and 5. In Experiment 1, the estimated modulation was 0.045 (arbitrary units) (95% HDI: [−0.015, 0.102]) and the posterior odds in favor of a positive modulation were 14.0:1, indicating evidence for the expected source-level reversal. In Experiment 2, the corresponding profile modulation was smaller and more uncertain: the estimated modulation was 0.017 (95% HDI: [−0.033, 0.067]) and the posterior odds in favor of a positive modulation were 3.1:1, indicating only weak evidence for a reversal pattern. We note, however, that the source estimation was coarse and, given the lack of subject-specific MRIs and electrode digitization, the results should be interpreted with caution.

Finally, we performed frequentist t-tests (two-sided) to compare horizontal gaze bias between the two experiments in each condition. The results were as follows: condition 1: $t(43) = -0.94$, $p = 0.35$; condition 2: $t(43) = -0.32$, $p = 0.75$; condition 3: $t(43) = 0.98$, $p = 0.33$; condition 4: $t(43) = -0.10$, $p = 0.92$; condition 5: $t(43) = 1.0$, $p = 0.3$. Bayes factors for these tests all favored the null hypothesis ($BF_{01} \geq 2.16$).
